# Morphologic and Aerodynamic Considerations Regarding the Plumed Seeds of *Tragopogon pratensis* and Their Implications for Seed Dispersal

**DOI:** 10.1371/journal.pone.0125040

**Published:** 2015-05-04

**Authors:** Vincent Casseau, Guido De Croon, Dario Izzo, Camilla Pandolfi

**Affiliations:** 1 Advanced Concepts Team, European Space Research and Technology Centre, Noordwijk, The Netherlands; 2 Micro Air Vehicle Laboratory, TU Delft, Delft, The Netherlands; 3 Department of Agrifood Production and Environmental Sciences, University of Florence, Firenze, Italy; University of Western Sydney, AUSTRALIA

## Abstract

*Tragopogon pratensis* is a small herbaceous plant that uses wind as the dispersal vector for its seeds. The seeds are attached to parachutes that increase the aerodynamic drag force and increase the total distance travelled. Our hypothesis is that evolution has carefully tuned the air permeability of the seeds to operate in the most convenient fluid dynamic regime. To achieve final permeability, the primary and secondary fibres of the pappus have evolved with complex weaving; this maximises the drag force (i.e., the drag coefficient), and the pappus operates in an “optimal” state. We used computational fluid dynamics (CFD) simulations to compute the seed drag coefficient and compare it with data obtained from drop experiments. The permeability of the parachute was estimated from microscope images. Our simulations reveal three flow regimes in which the parachute can operate according to its permeability. These flow regimes impact the stability of the parachute and its drag coefficient. From the permeability measurements and drop experiments, we show how the seeds operate very close to the optimal case. The porosity of the textile appears to be an appropriate solution to achieve a lightweight structure that allows a low terminal velocity, a stable flight and a very efficient parachute for the velocity at which it operates.

## Introduction

Wind dispersed seeds have been extensively investigated [1–4]. Some of these seeds produce winged structures to gain some lift during gliding or autorotation; other seeds maximise drag by building hairy plumes [1]. The plumes (pappi) can be arranged in different shapes. In still air, the presence of pappi result in vertical descent. In a windy environment, seeds travel horizontally; the seeds benefit from lateral wind currents and achieve great distances. Empirical data support the basic intuition that the horizontal travel of a seed is inversely proportional to its terminal velocity [5]. Therefore, parachutes are designed to maximise drag and increase the horizontal flight capacity of seeds.

In an attempt to predict the relationship between fruit mass, pappus area and terminal velocity, the rate of descent was measured for many species [2, 3]. Direct observations confirm the physical modelling where the rate of descent increases linearly with the square root of the wing loading (the ratio of mass to the projected area) when different plumed seeds are compared. These results suggest how morphology can contribute to variations in performance [2].

Pappus seeds were also compared to winged seeds. At lower wing loadings, plumes were more “efficient” in reducing terminal velocity. This is because the drag coefficient rises sharply as the Reynolds number declines [6]. For larger loads, different designs (i.e., small wings) have evolved due to the constraints involved in building bigger structures. In order for larger seeds to evolve, a species must face the following mechanical problem [4]: if the physical dimensions of the plume and the mass of the seed are scaled isometrically, plume loading increases with the bending of the hairs [6].

In this study, we focused on *Tragopogon pratensis*. This species is exceptional in terms of performance. In fact, the plant produces plumed seeds with mass and size comparable to winged seeds [6].

*Tragopogon* is an herbaceous species of the *Asteraceae* family. Seeds have stalked parachutes that are thought to be the largest found in nature; the final size of the parachutes is achieved via plumed fibres that are arranged hierarchically. In comparison to plumed seed species such as *Taraxacum officinale*, *Tragopogon* plants invest in larger and heavier seeds to increase the likelihood of germination. However, a species that has been subject to selection for heavier seeds must adjust its architecture to increase the size of the parachute such that low terminal velocity and good dispersal capacity can be maintained.

In Table 1, the performance of *Tragopogon* spp. with *Taraxacum officinale* is directly compared. Although they have similar shapes, the seeds of *Taraxacum* are approximately ten times lighter and consist of hundreds of unbranched hairs. Despite this difference, the terminal velocity of the two seeds is comparable.

**Table 1:** *Tragopogon* spp. and *Taraxacum officinale*, a comparison table.

Plant species	Mass (mg)	Pappus radius (mm)	Rate of descent (m∙s^-1^)	Ref.
*Taraxacum officinale*	0.62	6.2	0.269	[4]
	0.68	5.29	0.42	[2]
		4.47	0.65	[3]
*Tragopogon dubius*	2 to 14	15 to 29	0.2 to 0.6	[7]
		22.47	0.33	[3]
*Tragopogon pratensis*	11.20		0.57	[8]
*Tragopogon pratensis* subsp. *pratensis*	4 to 17			[9]
*Tragopogon porrifolius*	7.05 to 8.89	21.79 to 22.65	0.30 to 0.36	[2]

Fruit mass and terminal velocities of *Tragopogon* spp. and *Taraxacum officinale* reported in the literature.

This change in the size, shape and rate of descent is in agreement with the proportionality predicted by the following general aerodynamic assumption:

Terminal velocity massprojected pappus area 1

From a structural point of view, *Tragopogon* had to change the shape of the parachute to maintain performance comparable with *Taraxacum*. This was achieved by scaling the parachute up and introducing a second order of fibres and a supporting structure.

For large parachuted seeds, the conical parachutes of seeds from the genus *Tragopogon* have a remarkably different design. Some of the key features that determine their favourable aerodynamic performance are presented in this paper.

Our hypothesis is that the air permeability of *Tragopogon* parachutes evolved to operate in the most convenient fluid dynamic regime. This was achieved by arranging the fibres in a complex weaving to maximise the drag force (i.e., the drag coefficient) and operate in an “optimal” state.

To test our hypothesis, we used a fluid dynamics approach. In fluid dynamics, the drag coefficient *C*_D_ is one of the dimensionless quantities that are defined to describe how well the object performs. Accordingly, *C*_D_ depends on the shape of the object. The fibre arrangement determines the parachute permeability, which in turn is the main contributor to the seed drag coefficient. It is extremely difficult to test the effect of permeability on the aerodynamic properties of the seed through wind-tunnel experiments; therefore, we used a computational fluid dynamics (CFD) approach. We performed 2D and 3D CFD simulations to compute the seed drag coefficient and compare it to data calculated from seeds collected in nature. From a biomimetic point of view, the study of the permeability of *Tragopogon* could provide new insights into parachute design research; for example, it could be possible to design more efficient parachutes by tuning the drag-force simply changing the porosity of their fabrics; therefore, we decided to address this feature thoroughly.

## Materials and Methods

### Plant material and pappus anatomy

**Fig 1:**
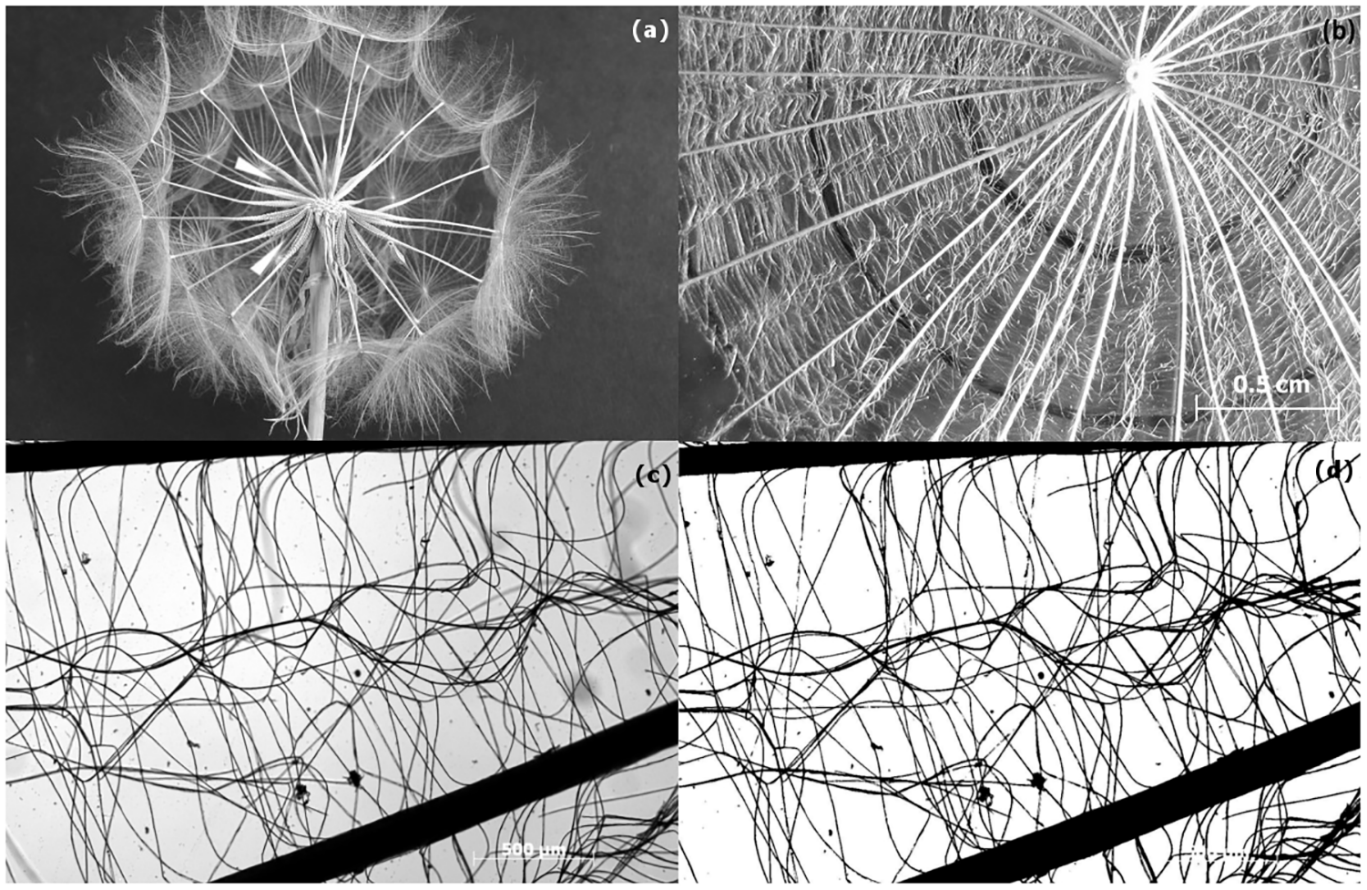
Details of the *capitulum* and the single parachutes of *Tragopogon pratensis*. (a) A collected *capitulum*; the white arrows point at the central and peripheral achenes, whose difference in size is evident. (b) A parachute unfolded between two glass slides. The black lines in the background indicate the three concentric regions analysed (0–5 mm; 5–10 mm; 10–15 mm). (c-d) Inverted microscope image of a portion of the parachute before (c) and after (d) the transformation to a binary image.

The plant material used for this work came from a natural population of *T*. *pratensis* growing in the area of Noordwijk, the Netherlands. No specific permissions were required to collect the material; in fact, *T*. *pratensis* is common wild flower that grows naturally along the roads, and it is usually regarded as a weed. Seeds were collected from 10 individuals from multiple populations in the late spring. Studies were conducted using pappi from natural dried fruits. The structure and anatomy of the hairs were studied by electron scanning microscopy (SEM), inverted bright field microscopy and direct measurements. The reported dimorphism of the achenes of *T*. *pratensis* [9] was also considered. Central and peripheral seeds were separated (Fig 1a), the parachutes were analysed and the achenes weighted (10 achenes from each type were collected from the *capitula* of 5 different plants). For the optical microscope analysis, the conic parachute was unfolded and placed between two glass slides (Fig 1b). To collect SEM images, the samples were platinum coated and observed using different magnifications. Time-lapse imaging has been used to record the closing of the parachute when wet. A drop of water was placed in the middle of the parachute, and the rapid closing and reopening was filmed (at a sampling interval of 30 s).

The main morphological parameters of the seeds were calculated from the collected images using the image-processing program ImageJ (U. S. National Institutes of Health, Bethesda, Maryland, USA).

Statistical analysis of data was processed using analysis of variance (one-way ANOVA and t-test). For ANOVA, differences between columns were assessed using Tukey’s Multiple Comparison Test. Differences were considered significant if P < 0.05. All the tests were performed using the software Graph-Pad Prism (Ver. 6.0 for MAC OS X).

### Terminal velocity measurements and the computation of *C*_*D*_

Five randomly selected seeds from each group were dropped in a closed room to minimise air turbulence. Movement was filmed using a Full HD Sony Handycam (10.2 megapixels) mounted on a tripod. Each seed was dropped ten times and recorded over 1 m. The terminal velocity was calculated as follows. Seeds were dropped at approximately 1 meter from the camera. A camera calibration was performed to remove the distortion caused by lens effects such that the images were linear projections corresponding to a pinhole model, as previously described [10]. For the velocity determination, the undistorted images were automatically processed. First, a robust background-based motion detection algorithm extracted the pixels of moving objects in the video images. Noise was then removed. Due to size selection, only large regions of motion corresponding to the single falling seed were retained. The coordinates of this region in the image were represented by a box that contained the seeds. Therefore, the size and the position of the seed could be determined automatically over time (S1 Video; see supporting information with the online version of this article). The only assumption was that the seed had a limited rotation/tilt, as is the case for the free falling seeds in the still environment of the experiment. The size of the box was used to determine the distance from the seed to the camera, given that the rib size of each seed was known. The velocity was estimated based on the entire vertical trajectory of the seed, with the help of a robust linear fit.

The *C*_D_ was derived using the following equation:

$$F_{D}=\frac{1}{2}\rho v^{2}AC_{d}$$ 2

where F_D_ is the drag force, *ρ* is the air density, V_∞_ is the speed of the object relative to the fluid, *A* is the cross-sectional area of the object, and *C*_*D*_ is the drag coefficient, which is a dimensionless number.

The mass, *m*, and the pappus area, *A*, was measured for each seed. The drag force is simply rewritten as the product of *m* by the standard gravity, *g*, which is equal to 9.81 m∙s^-2^. Therefore, the calculation of the drag coefficient is straightforward.

### Porosity measurement

The porosity variable *E* is a geometric parameter of a body. It is commonly defined as the ratio of void volume to the total volume of the medium. In the present case, the geometry is the supposed invariant in the direction normal to the medium surface. Porosity thus becomes a ratio of areas. Following Green and Johnson [6], porosity is the ratio of the total projected area of the void spaces to the total plan area of the imaginary disk formed by the pappus.

$$E_{hairs}=\frac{A_{white\;px}}{A_{total\;px}}$$ 3

To characterise the porosity along the parachute, the pappus surface was divided into three concentric areas according to the distance from the centre (0–5 mm, 5–10 mm and 10–15 mm). For this purpose, inverted microscope images were used (6 mm^2^ per image, with a resolution of 2 μm/pixel; Fig 1c). Each image was transformed in a binary image (with hairs in black and the background in white; Fig 1d). The black and white pixels were counted to calculate the ratio of white pixels, *A*_white px_, to the total number of pixels, *A*_total px_. At such a high magnification, the portions of the primary ribs included in the images dramatically affected the porosity. Therefore, *E*_hairs_ was calculated in the portions of images entirely composed of secondary hairs. The contribution of the ribs to the total porosity was later estimated as follows:

$$S_{ribs}=\frac{\left(D_{I}+D_{III}\right)\times L}{2}$$ 4a

$$S_{hairs}=\left(S_{cone}-S_{ribs}\right)\times \left(1-E_{hairs}\right)$$ 4b

$$E=1-\frac{S_{hairs}+S_{ribs}}{S_{cone}}=E_{hairs}\left(1-\frac{\left(D_{I}+D_{III}\right)\times L}{2S_{cone}}\right)$$ 4c

where *S*_ribs_ and *S*_hairs_ are the surfaces occupied by the ribs and the hairs, *D*_I_ and *D*_III_ are the diameters of the ribs measured in the first and third regions from the centre, respectively, *L* is the total length of the ribs, and *S*_cone_ is the total surface area of the parachute.

### Permeability

Air permeability is a very important physical quantity in the study of aerodynamic performance of textile materials. For example, it significantly affects the drag coefficient. Air permeability is defined as the rate of airflow through a material under differential pressure [11] and is mainly dependent on the thickness, structure, porosity, and the makeup of the fabric. Air permeability is typically measured experimentally. Given the very small thickness of the parachute, assessing permeability experimentally was unfeasible. Consequently, we determined the permeability by applying the Kozeny-Carman equation [12, 13], as proposed by Knauf and Doshi [14] for materials that have a similar thickness. This equation relates the air permeability, *K*, to the porosity of the medium [15, 16] as follows:

$$K=\frac{E^{3}}{kS_{v}^{2}{\left(1-E\right)}^{2}}$$ 5

where *k* is Kozeny factor, *S*_*v*_ is the fibre surface area per unit volume of fibre, and *E* is the porosity. The Kozeny factor was estimated using Eq (6) as described previously [14]. This equation is a function of porosity and holds for a porosity range of 0.68–0.96.

$$k=5+10^{\left[14\left(E-0.8\right)\right]}$$ 6

Achieving a satisfactory formulation to determine *S*_*v*_ was challenging. It is simply not possible to translate the intricate weaving of the secondary hairs into a mathematical expression without any simplification. We therefore represented the primary and secondary hairs as cylinders, with diameters *d* and *D*, respectively, that form a homogeneous medium. *S*_*v*_ can now be calculated using the following equation:

$$S_{v}=\frac{fiber\;surface\;area}{unit\;volume\;of\;fiber}=\frac{\left(ld\pi \right)+\left(LD\pi \right)}{l\pi \frac{d^{2}}{4}+L\pi \frac{D^{2}}{4}}=4\frac{dl+DL}{d^{2}l+D^{2}L}$$ 7

where *L* and *l* are the total length of primary and secondary hairs, respectively. *L*, *D*, and *d* were computed using the images of the parachute taken by the inverted microscope; *l* was obtained using indirect computations.

The area occupied by secondary fibres, designated *S*_hairs_, was calculated using Eq (4b). The fibres were considered to be long cylinders, whose areas were projected on the surface of the parachute. The total length of the secondary fibres, *l*, is estimated as the ratio of *S*_hairs_ to the mean diameter *d*.

### Reynolds number

The Reynolds number, *Re*, is the ratio of inertia to viscous forces. The laminar or turbulent nature of a flow moving over a body is characterised using the Reynolds number. Following the methodology described in Rhodes [17] and taking into account the porous texture of the fibres, *Re* was estimated to be approximately 2. For porous media, a fully laminar condition exists at a *Re* less than 10. Therefore, all our computations were performed under this assumption (S1 Appendix).

### Computational Fluid Dynamics simulation

For a first study of the aerodynamic properties of seeds that introduces the porosity of the material as a key quantity, it seems irrelevant to reproduce the arrangement of the fibres as closely as possible. Some simplifications were made to define the physical bounds of the body. The parachute was modelled as a cone, and primary and secondary fibres were replaced by a surface with an assigned permeability. This simplification did not generate a computationally time-efficient 3D model that was satisfactory for use in our tests. A further simplification took advantage of the rotational symmetry of the conical geometry of the seed. This second model is referred to as the 2D axisymmetric cone. In this scenario, the physical domain is restricted to a plane whose bottom boundary is the revolution axis. All of the details of the CFD simulations are given in the supporting information (see S1 Appendix).

The complexity of the 3D model is such that the duration of a simulation can be up to 2 weeks. In contrast, the 2D simulation can run in few minutes. For this reason, the 2D axisymmetric model was used as a quick way to assess the downstream flow dynamics for a wide range of permeability values, cone angles and free-stream velocities. A few 3D simulations, which were sometimes more accurate, were used to validate the aforementioned results. The consistency of the results between the 2D and 3D simulations was assessed by analytically comparing the drag coefficients, pressures and velocity fields in the entire fluid domain.

Eventually, the flow-field obtained for each simulation was used to compute the aerodynamic drag force acting upon the pappus; its drag coefficient could then be determined (S1 Appendix). If not otherwise specified, computations were conducted with a free-stream velocity equal to 0.3 m∙s^-1^, which was the mean terminal velocity recorded from our experiments, a 22-degree cone angle, and a parachute of constant permeability with a thickness of 100 μm.

## Results

### Morphological analysis of the parachute

**Fig 2:**
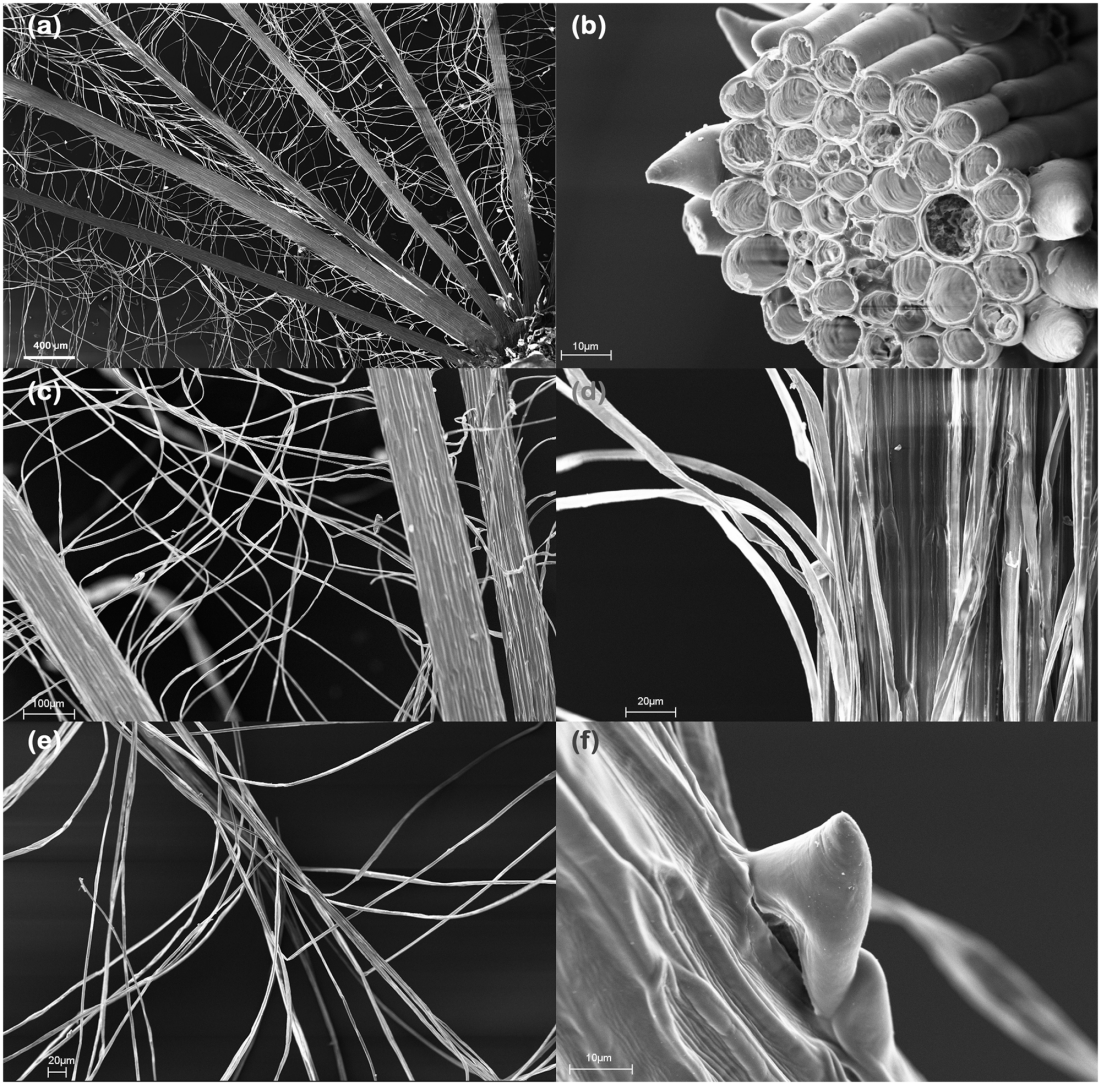
Scanning electron images of the parachute. (a) Hierarchical arrangement of the fibres; (b) Cross-section of a primary rib showing its internal hollow structure; (c-e) Details of the complex weaving of the secondary fibres; (f) Hook at the end of the rib that may serve as a means of secondary dispersal.

As shown in Fig 2a, the parachute of *T*. *pratensis* consists of two orders of fibres. The main bundles of cells, which mechanically support the structure, are referred to as the “primary” hairs or ribs. The more slender cells, which create the fine textile, are referred as “secondary” hairs or fibres. Primary hairs have a filamentous structure with a mean length of 18.43 mm and mean diameter of 113.3 μm. Several fibre cells constitute the primary hairs (Fig 2d). In a transverse section, it can be observed that the cells are arranged regularly. When the seed dries, the fibre cells create a lightweight structure made up of hollow tubes (Fig 2b). The secondary fibres are individual cells that partially detach from the main ribs and tangle together to create fine and complex weaving (Fig 2c). Secondary fibre cells have a mean diameter of 5 μm and detach from the two sides of the ribs. The fibres that come from two adjacent ribs intersect in the middle to create a central line (Fig 2e). This provides a homogeneous porosity that overcomes the radial geometry of space. Spines are present at the end of the main ribs (Fig 2f) and may serve as an additional seed dispersal method. All of the morphological parameters are reported in Table 2. Concerning the morphology of the parachutes, no significant differences between the values for central and peripheral parachutes were found. The data presented in the table are the means calculated for the data pulled from both types of parachutes. These mean values were then used for the model. Concerning the dimension of the ribs, given the presence of the spines, an effective dimension of 15 mm was considered for the model.

**Table 2:** Morphological and aerodynamical parameters of the collected seeds from *Tragopogon pratensis*.

*Achene and parachute masses (Mean*, *SE n = 10 p<0*.*05)*	Peripheral	Central	
Parachute *(mg)*	203 ± 0.31	1.99 ±0.4	ns
Achene *(mg)*	9.29 ± 0.25	588 ± 0.2	***
Total mass *(mg)*	11.32 ± 0.27	7.87 ± 0.2	***
% mass allocated in the parachute	18%	25%	-
*Aerodynamical parameters*	Peripheral	Central	
Terminal velocity of the parachutes (m s^-1^)	0.336 ± 0.013	0.270 ± 0.003	***
Drag coefficient C_D_	1.15 ± 0.04	1.26 ± 0.07	ns
*Mean Parachute morphology used for the permeability assessment (Mean*, *SE n = 20)*			
Main rib length (mm) ^*(*^ ^*a*^ ^*)*^	18.43 ± 0.72		
Number of ribs^*(*^ ^*a*^ ^*)*^	27.6 ± 0.60		
Rib inclination in the horizontal plane (angle) ^*(*^ ^*a*^ ^*)*^	21.01 ± 2.28		
Main rib mean diameter (μm) (*D*) ^*(*^ ^*a*^ ^*)*^	91.80 ± 18.62		
0–5 mm (*D*_I_)	102.76 ± 16.42		
5–10 mm(*D*_II_)	92.85 ± 17.48		
10–15 mm(*D*_III_)	79.79 ± 15.18		
Secondary fibre diameter (*d*) (μm)	5 ± 0.33		

The measured parameters for the central and peripheral seeds are reported in the table.

^(a)^ As there were no significant differences between the values for the central and peripheral parachutes, the data presented in this table are the means calculated for the data pulled from both types of parachute. These mean values were then used for the model.

**Fig 3:**
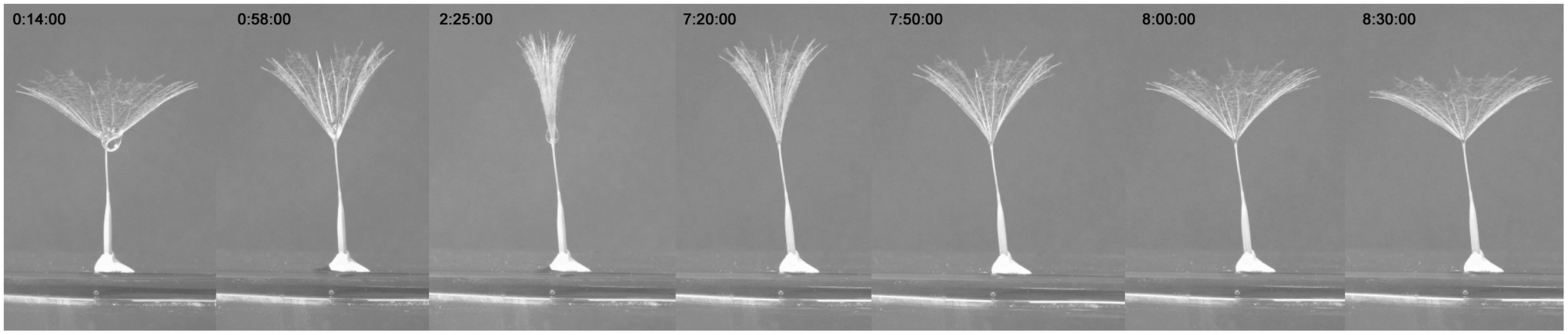
Parachute closure due to changes in humidity. When a drop of water is placed at the centre of the parachute, it rapidly closes; the parachute resumes its original status when it is dry again.

The rib inclination in the horizontal plane determines the cone angle of the structure and has an impact on the aerodynamics of the parachute. It ranges from 0 degrees, when the parachute is wide open to form a planar disk, to 90 degrees, when the parachute is completely folded. For freshly collected seeds, this angle has a mean value of 22 degrees. From field observations, we noticed that the parachutes responded to changes in external humidity for few days after the first opening of the c*apitulum* and adjusted the cone angle accordingly. Therefore, rain or dewdrops result in parachute closure. Once dried, parachutes recover their original configuration. A deep investigation of this mechanism was beyond the scope of this study; therefore, only few experiments were performed to observe this phenomenon. Reversible movement was recorded in a time-lapse video, which is shown in Fig 3 and in S2 Video. When a drop of water is placed at the centre of the parachute, the sudden closure of the parachute occurs. The parachute recovers its final opening angle as soon as the central active region is dry.

### Terminal velocity measurements and computation of *C*_*D*_

The measured terminal velocities and drag coefficients for peripheral and central parachutes are reported in Table 2. The heavier peripheral seeds fall significantly faster than the central ones. Although light seeds have higher drag coefficients than heavy seeds, the *C*_*D*_ of the two groups are not statistically different, probably due to the small sample size.

### Porosity and permeability range of the parachute

Following the methods above, the porosity of the parachute was calculated. The density of the secondary fibres does not change with the radius (Table 3). The different porosity of the three regions is determined by the radial presence of the primary ribs. For this reason, we assumed a mean porosity along the entire structure in the CFD simulations.

**Table 3:** Porosity and permeability values for the total area and for the three concentric regions of the parachutes.

	Mean	0–5 mm (*D*_I_)	5–10 mm (*D*_II_)	10–15 mm (*D*_III_)
Porosity of the secondary hairs^(^ ^a^ ^)^	0.956 ± 0.018	0.957 ± 0.005 ns	0.955 ± 0.006 ns	0.956 ± 0.003 ns
Total porosity	0.905 ± 0.009	0.787 ± 0.028	0.905 ± 0.010	0.929 ± 0.005
Mean Permeability [m^2^]	1.59 10^–9^	-	-	-
Permeability Range [m^2^]	5.77 x 10^–10^ < *K* < 4.41 x 10^–9^	-	-	-

^(a)^ n = 15 sections from 3 parachutes *p<0*.*05*.

The porosity was translated into a permeability value using the Kozeny-Carman equation. As shown in Table 3, the mean permeability of the seeds was found to be 1.59 x 10^–9^ m^2^. We also defined a permeability range within which the seed can operate that accounts for the natural variation of the rib diameter and the porosity of the secondary fibres. There is no calculation of this sort in the literature. In terms of aerodynamics, the pappus has always been considered to be a non-porous body; this approach greatly simplifies scientific reasoning and problem solving.

### Flow regimes

**Fig 4:**
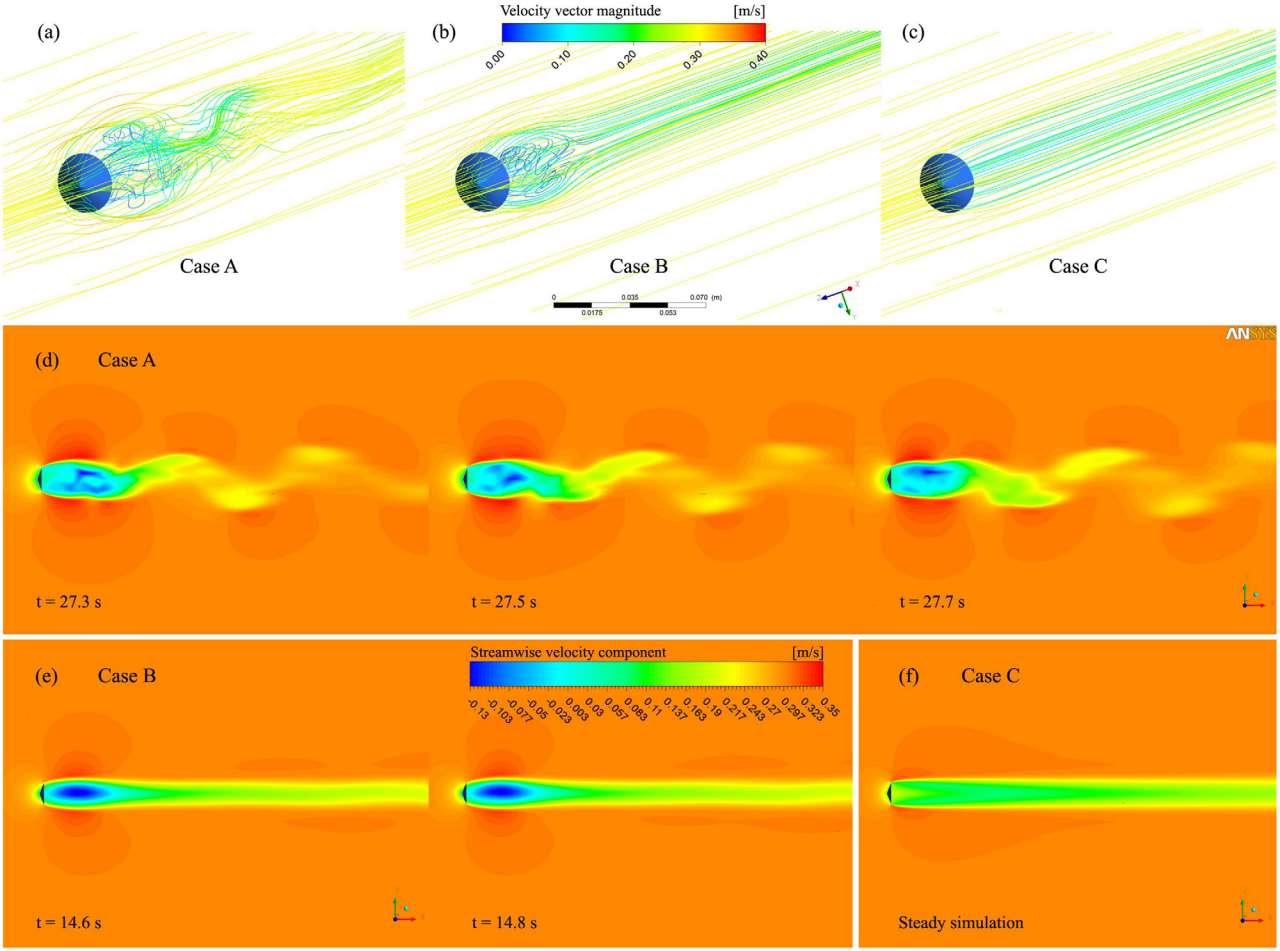
Mathematical modelling. (a-c) Streamlines for the three flow fields observed in the reference case. By controlling the rate of airflow passing through the parachute, the permeability greatly influences the flow dynamics in the wake region. (d-f) The stream-wise component of the velocity vector is reported for the three case scenarios in 3D computations. The cone is represented in black, and the airflow moves from left to right. Case A—An unsteady vortex flow, for *K* = 1.0 x 10^–9^ m^2^, is shown at three time steps. The vortex shedding phenomenon is clearly visible (a, d); Case B—A steady vortex flow for two distinct time-steps (b, e). The negative values for the stream-wise velocity component just downstream the body (blue region) represent the recirculation area; Case C—Laminar shear flow for *K* = 6.0 x 10^–9^ m^2^ (c, f).

Our 3D simulations revealed that different fluid regimes develop according to the permeability values assigned to the parachute; these permeability values range from 1 x 10^–10^ to 5 x 10^–6^ m^2^. This is similar to what known about low Reynolds numbers in the case of non-permeable cylinders. In particular, three fluid regimes have been identified. The three cases (Case A, Case B, and Case C) for a free-stream velocity of 0.3 m∙s^-1^ are depicted in Fig 4 and described below.

*Case A*—For a permeability close to a “wall” situation (*K* < 3.8 x 10^–9^ m^2^), the flow regime observed in the wake of the parachute is complex and characterised by the presence of vortices (Fig 4a, S3 Video). The position and shape of the vortices fluctuate over time (Fig 4d, S4 Video). The unsteady, oscillating flow in this case scenario is called vortex shedding and is likely to generate instabilities [18]. *Case B*—A further decrease in the permeability of the medium (3.8 10^–9^ m^2^ < *K* < 4.2 x 10^–9^ m^2^) results in a weak recirculation of air downstream. A steady axisymmetric vortex is present in the wake (marked in dark blue in Fig 4b–4e). *Case C*—When *K* is greater than 4.2 10^–9^ m^2^, a sufficient flow rate allows air to easily pass through the fibres. The wake of the body takes the form of a laminar shear layer. It is a steady-state flow, presenting no vortices in the wake region (Fig 4c–4f).

### Drag coefficient

**Fig 5:**
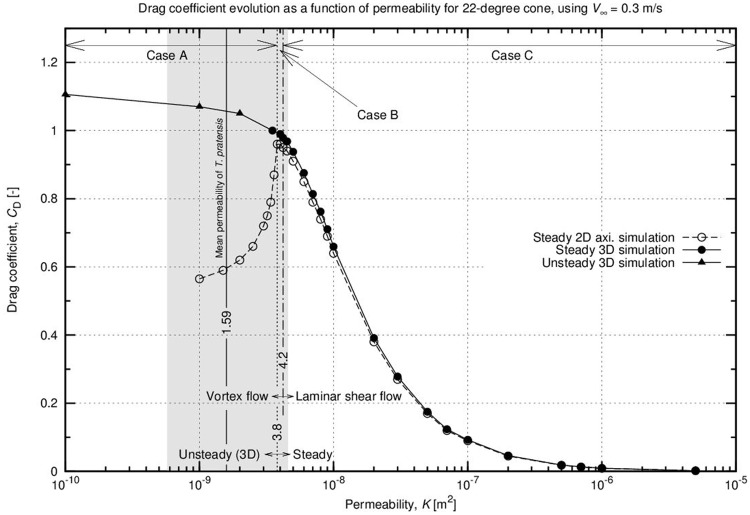
CFD simulations for 2D axisymmetric and 3D cones, a reference case. Computations are run with steady (circles) or unsteady (triangles) flow hypotheses. Filled symbols represent tri-dimensional computations and are associated with the solid line. Unfilled symbols represent simulations run under axisymmetric assumptions and are connected with a dashed line. Both the solid and dashed lines show that the trend of the drag coefficient is a function of the permeability; the dashed line is constant along the radius of the cone and 22 degrees in angle. The free stream velocity is set to 0.3 m∙s^-1^.

The evolution of the drag coefficient as a function of parachute permeability for both the 2D and 3D computations is presented in Fig 5. Permeability is assumed even along the cone radius and ranges from 1 x 10^–10^ to 1 x 10^–5^ m^2^. The *C*_*D*_ values obtained from the CFD simulations are comparable with those obtained from the drop experiments in Table 2.

As explained above, the 3D computations are closer to reality; however, we also run 2D simulations due to time constraints. Both the 2D and 3D computations are plotted as a matter of comparison. The two curves overlap for permeability larger than 4.2 x 10–9 m2. For smaller values, the drag coefficients do not match. In the 3D computations, a change in the slope occurs at 4.2 x 10–9 m2; a peak is located at the same place in the 2D computations. In light of the 3D model results, the peak observed in the 2D model can be interpreted as a loss of axisymmetricity in the flow field. This peak represents an artefact of the CFD computations for a cone modelled under axisymmetric assumptions. For permeability values greater than the peak, the difference in CD between the two models is less than 3%. In light of these considerations, the 2D axisymmetric model is a useful and quick screening tool to determine the aerodynamic performance of the cone shaped parachute over this permeability range.

As shown in Fig 5, the drag coefficient in the 3D model is at its highest for permeability approaching zero. *C*_*D*_ decreases smoothly up to *K* = 4.2 x 10^–9^ m^2^. The flow regime is characterised by unsteady vortices (Case A). In this region of the curve, a decrease in the plant investment in the fabrication of the parachute (i.e., an increase in permeability) does not have a strong impact on its efficiency; *C*_*D*_ does not decrease significantly. When a steady shear flow regime is present (Case C), the opposite situation is observed; a decrease in the density of the hairs results in a sharp decrease in parachute efficiency. An intermediate situation occurs in Case B. The unsteady vortices shown in Case A progressively lose their intensity and eventually fade away when *K* approaches 4 x 10^–9^ m^2^. This results in a steady air recirculation in the near-wake region. The grey region of the graph corresponds to the permeability range of *Tragopogon* seeds as computed in the previous section, describing the flow regime at which the majority of the seeds are operating.

### The influence of cone angle and free-stream velocity on the drag coefficient

**Fig 6:**
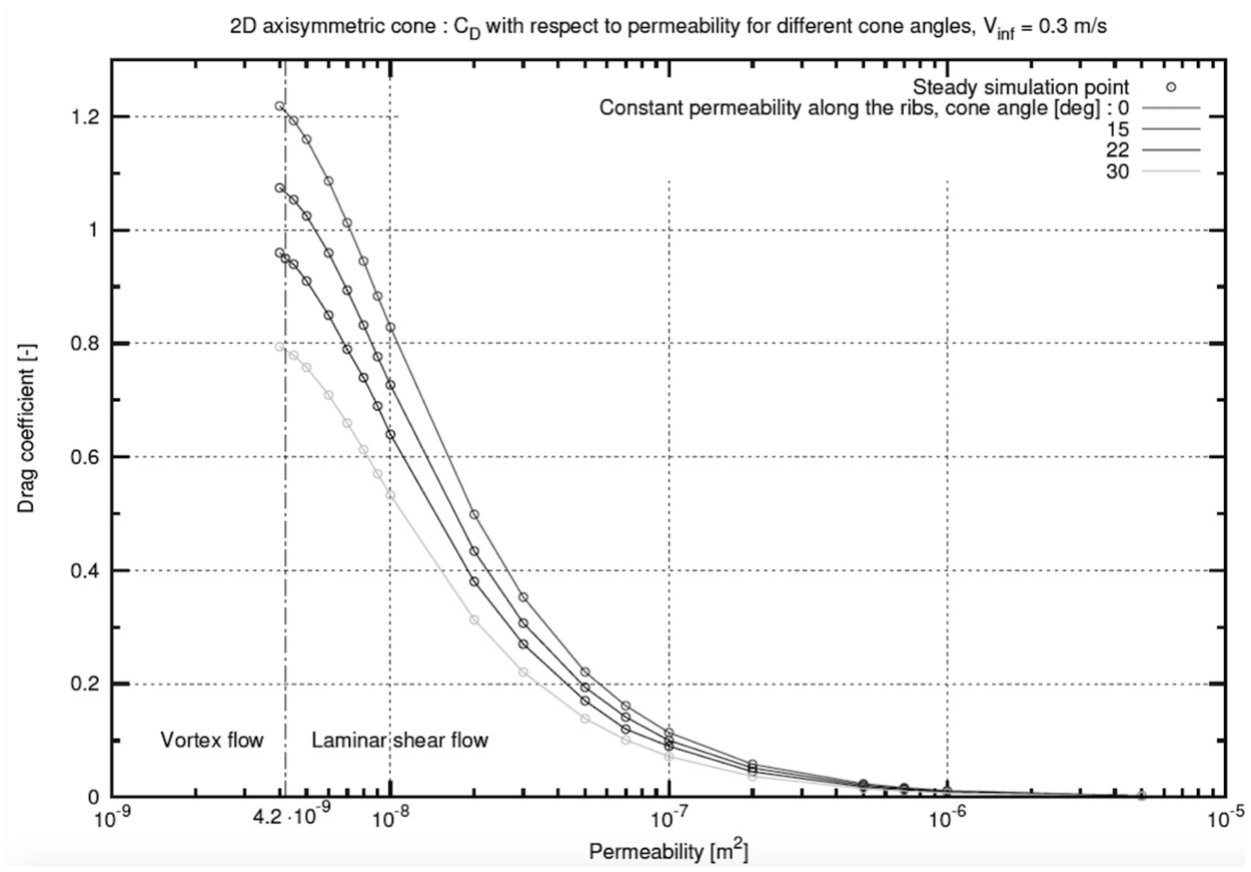
CFD simulations for 2D axisymmetric cone. Influence of the angle of the cone on drag coefficient as a function of permeability.

To assess how *C*_D_ is affected by changes in the cone angle, we ran 2D simulations for the following cone angles: 0, 15, and 30 degrees. The trend observed for *C*_D_ is similar to the one observed for the 22-degree reference. Greater drag coefficients are achieved for lower cone angles (Fig 6). We also noted that the transition from vortex flow to laminar shear flow occurs at the same permeability value independent of the pappus angle.

**Fig 7:**
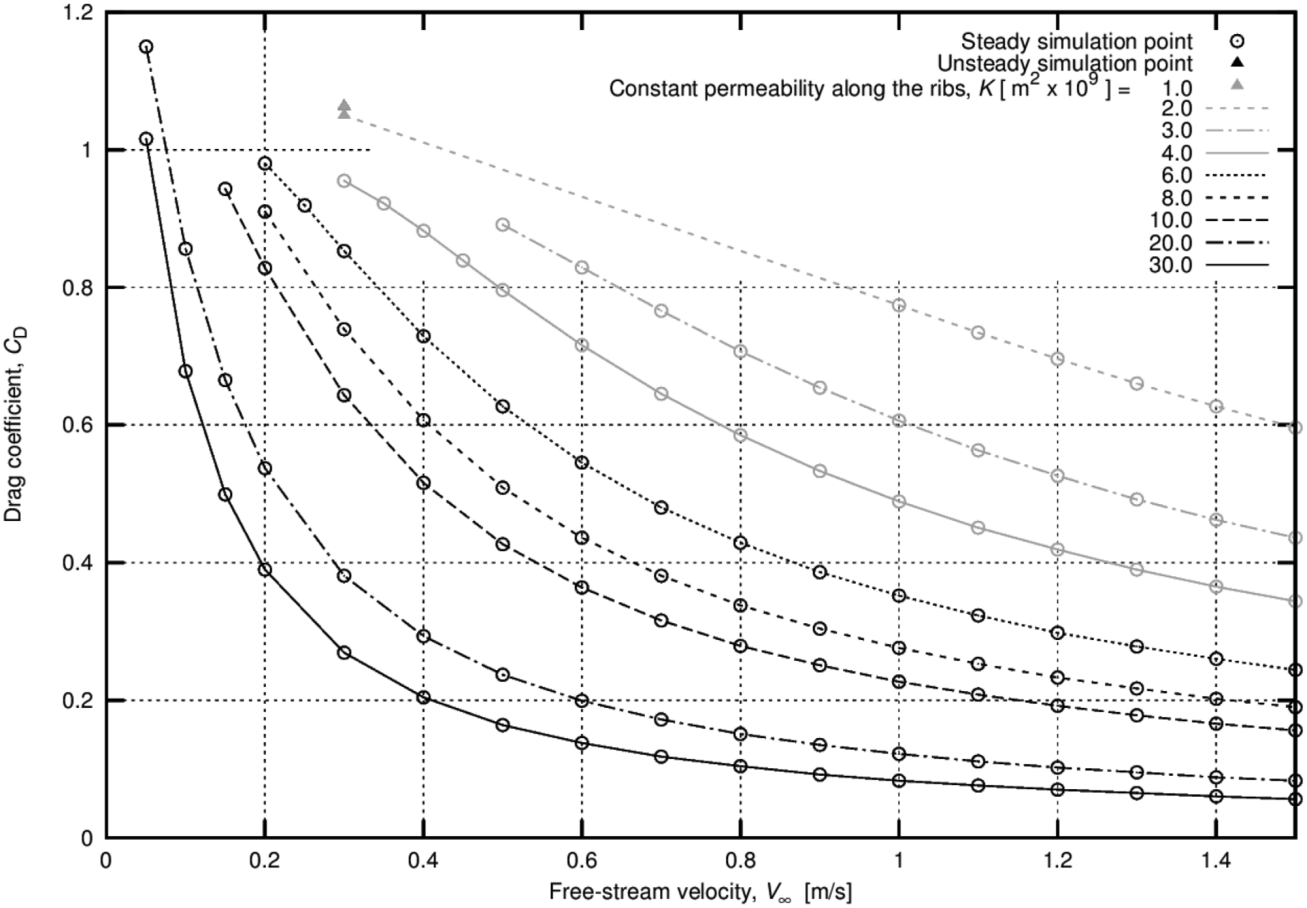
The influence of the free stream velocity (and the Reynolds number) on the drag coefficient for different permeability values. The higher the permeability of the medium, the more pronounced the decreasing trend of the drag coefficient. For permeability equal to 2.0 x 10^–9^ m^2^, the curve is almost a straight line (the dashed line is a linear extrapolation of the results).

The weight of the achenes has a direct influence on terminal velocity. In our experiments, the mean terminal velocities of the central achenes and the peripheral achenes were 0.27 m∙s^-1^ and 0.33 m∙s^-1^, respectively. Therefore, we tested the effect of free-stream velocity on the drag coefficient. It appears that the transition from Case A to Case B occurs at a lower permeability when the terminal velocity is reduced. For a given permeability, drag coefficients are greater for lower terminal velocities (S1 Fig). Generally speaking, the drag coefficient varies dramatically as a function of the free-stream velocity and as a function of the Reynolds number (Fig 7).

## Discussion

### Aerodynamic performance of the seeds

Pappus seeds are very “efficient” in reducing terminal velocity at low wing loadings [6]. Different designs, such as winged seeds, have been evolved to support larger loads. In this paper, we focused on *Tragopogon*; the parachutes of *Tragopogon* seeds represent an exception to this phenomenon. These seeds have large cone-shaped parachutes, which are likely to be the largest reported in nature. The parachutes of *Tragopogon* seeds operate optimally at high wing loadings (comparable to those of winged seeds). *Tragopogon* seeds provide an exceptional case study in how evolution overcame the constraints involved in building larger plumed structures and the challenges derived from mechanics and aerodynamics.

SEM images of the seeds revealed a lightweight structure made of hollow fibres that were arranged in bundles to create a supporting skeleton. Cells detach from the bundles to create a thin, intricate fabric that forms the parachute itself. Although the hierarchical distribution of these fibres can be observed in many other pappus seeds (such as *Cirsum vulgare* and *Cynara cardunculus*), the arrangement of the fibres in *Tragopogon* seeds creates a complex textile that appears to be rather unique. The two orders of fibres allows the structure to be solid, thanks to the main ribs, and sufficiently permeable, thanks to the small fibres, resulting in a permeable, solid and lightweight structure.

From our results, it appears that the arrangement of the fibres has been optimised during evolution. The fibres determine the air permeability and, thus, the drag coefficient. In our numerical experiments, we explored a wide range of permeability and the effect of permeability on the aerodynamics of the system. We observed three specific cases in which the parachute can operate (see Fig 4). In Case A, the vortices in the wake keep the drag coefficient high. This configuration has two major downsides. The consistent amount of fibres that the plant has to build to achieve low permeability is one challenge. In addition, the vortices cause potential flight instabilities and unintended surface vibrations. The opposite situation is detectable in Case C; in this case, a laminar shear flow is observed. This determines a low *C*_D_, a stable flight, and a lower plant investment in the fibres. In light of these considerations, the optimal fluid regime is represented by an intermediate situation. In Case B, the presence of air recirculation significantly contributes to a loss in momentum. A reasonable *C*_D_ is achieved, but no unsteady vortices are observed in the wake of the parachute. In the present case, this particular regime is observed only in a very narrow window of permeability values (3.8–4.2 x 10^–9^ m^2^). As shown in Fig 5, the permeability range estimated for the parachute of *Tragopogon* embraces Cases A and B; Case B represents its lower limit. We hypothesise that optimisation in the reduction of fibres has ceased very close to the transition between Cases A, B, and C. Due to the fine tuning of fibre arrangements, the flight dynamics of seeds are very close to the optimal compromise between a high *C*_D_ value due to a low permeability and a relatively stable flight due to the low intensity of the vortices convected in the wake region.

### Seed morphological variation and the influence on the drag coefficient

The collected seeds had the following two main sources of variation: the cone angle and the achene mass. The cone angle is more or less constant among the parachutes stacked in the *capitulum*. However, the cone angle varies according to external humidity. This feature has been described for many wind-dispersed seeds. In *Salix* and *Populus* seeds, it has been reported that the hairs spread when dry and converge when wet; the *capitulum* of *Asteraceae* hides or exposes fruits through the movement of involucre bracts according to the amount of moisture [19]. However, this feature has not been previously reported for the opening of the single plumes of seeds from the genus *Tragopogon*. Here, we conjectured that such a mechanism serves as an additional strategy to control the time of seed dispersal. Two distinct effects create a greater force on the pappus when the cone angle is wide open (in dry conditions). The first effect is purely geometrical; the area offered to the wind is greater and produces a greater drag force. A further effect comes from the increased drag coefficient, which has been computed and reported in Fig 6 for various cone angles (*C*_D 30_ is 25% smaller than *C*_D 0_). As detailed in Eq (2), the drag force is linearly dependent upon the projected area A and the drag coefficient *C*_D_. An increase in the drag force with the cone angle is super-linear, helping the plant achieve its goal. The ability to bias seed dispersal in the most favourable conditions has been reported for many anemochorus species; a biased abscission mechanism has been previously described for *Tragopogon* [20].

Concerning the variation observed in the achene mass, it was determined that the seeds of *Tragopogon* are dimorphic. Lighter achenes are placed close to the centre; heavier achenes are found at the periphery of the *capitulum* [9, 21]. Considering that the parachute dimensions remain unchanged, the two configurations are subject to different disk loadings. In particular, a heavier seed will result in a higher disk load and tend to fall with a greater terminal velocity (Eq (1)). A greater terminal velocity will, over the permeability range we estimated for the parachute, have a significant effect on the drag coefficient. This was demonstrated in the computation of *C*_D_ values from the two groups of seeds. This effect further increases the terminal velocity, and seed dimorphism is leveraged.

Van Mölken et al. [9] showed that the achene size of *Tragopogon pratensis* seeds is the main factor determining germination percentage. This has also been reported for other non-dimorphic species [22–25]. Why *T*. *pratensis* produces dimorphic seeds is a question that remains to be answered. To maintain seed dimorphism, there must be an ecological difference between the morphologies [26]. In light of our aerodynamic studies, it can be argued that the central and peripheral seeds of *Tragopogon* plants have been optimised for two distinct tasks. Heavy peripheral achenes are meant to travel short distances, and central achenes are dedicated to longer travels.

Metapopulation models suggest that, in some landscapes, selection pressures against short-distance dispersal may lead to lighter seeds. In severely fragmented landscapes, reverse selection pressure can be found; seeds that disperse from the safe site can be considered lost [27]. *Tragopogon pratensis* can merge these two features; lighter seeds colonise new areas and heavier seeds substitute for the parental plant. A similar behaviour has been reported for the weed *Leontodon longirrostris* (*Asteraceae*). In *Leontodon*, the peripheral achenes are heavier and exhibit neither a pappus nor short-range dispersal; the central achenes are lighter, possess a well-developed pappus and are produced in greater numbers [28]. In *Leontodon*, the two types of achenes result in seedlings with differing vigour and ability to emerge from different achene burial depths. Corresponding data are unavailable for *Tragopogon*; however, it can be speculated that a similar strategy could be used in this species.

The uneven evolution of the drag coefficient for low Reynolds numbers in porous material has other interesting aspects. According to the work of McGinley and Brigham [7], terminal velocity is supposed to be proportional to the square root of disk loading *m*/*A*; this is under the implicit assumption that the drag coefficient is constant as a function of *Re*. It can be argued that this latter assumption is verified for a range of Reynolds number that refers to turbulent flows, which are some orders of magnitude greater than the values estimated for *Tragopogon* parachutes. Our computations reveal that the trend in drag coefficients, as a function of free-stream velocity, is in agreement with experiments for low Reynolds numbers, e.g. [3]). Indeed, the drag coefficient increases sharply when *Re* decreases (see Fig 7). This relates to the slower than predicted decline in terminal velocity observed when *A* is increasing, [7] under the assumption of *C*_D_ being constant.

Many attempts to characterise the aerodynamic properties of some flying seeds (including *Tragopogon*) can be found in the literature. Given the complexity of their shapes, only some approximations have been completed so far. For example, Greene and Johnson [6] modelled plumes as a bunch of cylindrical hairs that are attached radially to either the seed or a beak. The contribution of these hairs to the projected area of the plume was calculated under the assumption that hairs could be modelled as single long cylinders. In some species (i.e., *Taraxacum officinale* and *Asclepius syriaca*), this assumption resulted in successful analysis. However, the complex geometry of *Tragopogon dubius* (L.) failed to be correctly modelled using these assumptions.

In conclusion, *Tragopogon* represents a magnificent example of how a parachute has evolved to produce a drag force with interesting characteristics. Such a parachute allows for the efficient dispersal of the large achenes of this plant. In particular, the lightweight structure allows for a low terminal velocity, a stable flight and a very efficient parachute for the velocity at which it operates.

The technical challenges to be addressed in order to translate this design into an engineering prototype, would be to manufacture equivalent materials having the required porosity at different dimensions. Seed dispersal strategies are an overlooked and excellent source of inspiration for the development of new bio-inspired technologies, and the study of pappus seeds (also other than the *Tragopogon*) is likely to produce important insights for the design of more efficient parachutes.

## Supporting Information

S1 Video Automatic tracking for terminal velocity determination, the video is reproduced at normal speed; each square is 1 cm wide. (MOV)

S2 Video Timelapse of the reversible pappus closure. (MOV)

S3 Video Streamline for the flow regime in the wake of the parachute. (WMV)

S4 Video Stream-wise component of the velocity vector in the wake of the parachute. (WMV)

S1 Fig Influence of the free-stream velocity on drag coefficient as a function of permeability. (PDF)

S1 Appendix Reynolds number, CFD settings, computation of drag coefficient. (PDF)

S2 Appendix List of abbreviations. (PDF)

S1 Datasets Details of the morphological analysis, porosity measurements and sample images for porosity measurements. (ZIP)

S1 Archive Details of drop experiments: videos of the drops, txt file with the calculated averaged velocities. (ZIP)

S2 Archive Details of CFD simulations: 2D and 3D meshes, project file for 2D simulations, list of simulation runs, txt file with instructions to re-run the simulations. (ZIP)
