# Supplementary material for: Morphologic and Aerodynamic Considerations Regarding the Plumed Seeds of Tragopogon pratensis and Their Implications for Seed Dispersal
Source: PLoS One. 2015 May 4;10(5):e0125040. doi: 10.1371/journal.pone.0125040 (PMC4418730; doi:10.1371/journal.pone.0125040)
Supplement: S1 Fig — (PDF) [file pone.0125040.s005.pdf]

Figure S6

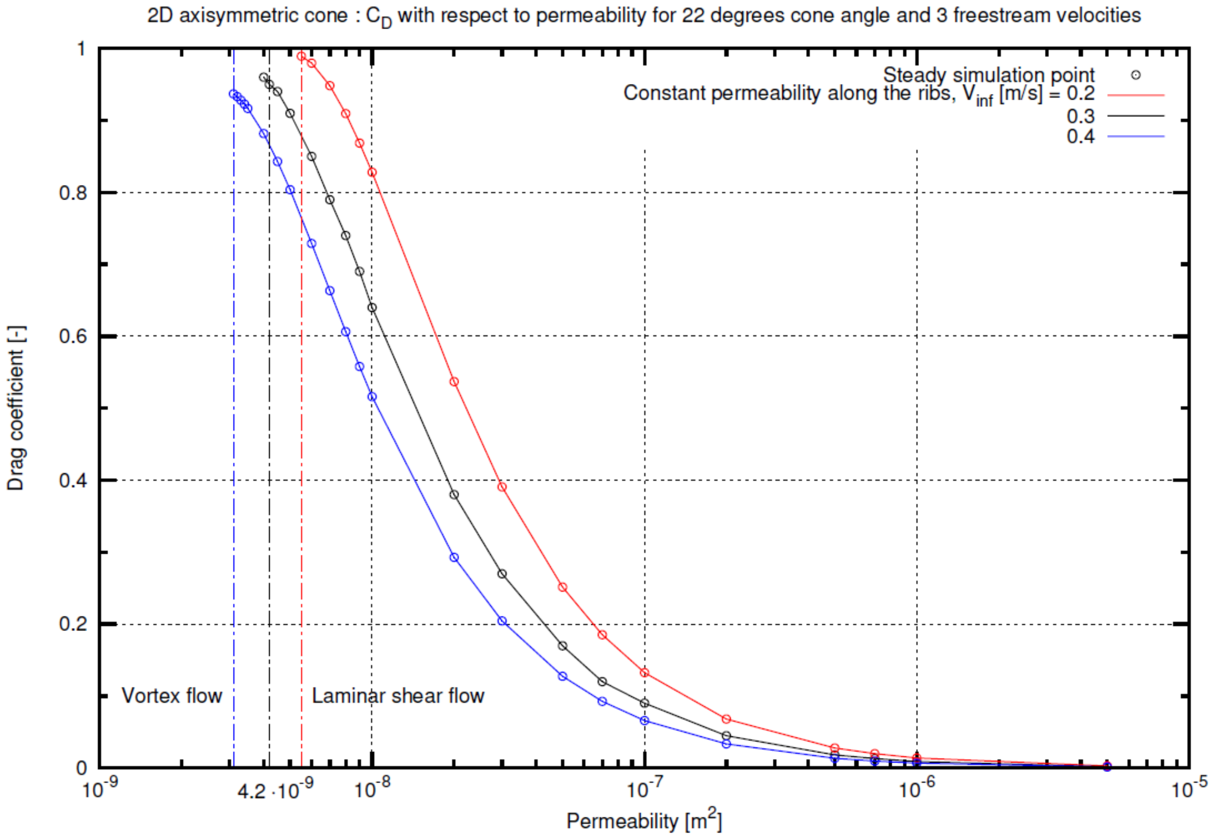

CFD simulations for 2D axisymmetric cone –Influence of the free-stream velocity on drag coefficient as a function of permeability.
