# Supplementary material for: Morphologic and Aerodynamic Considerations Regarding the Plumed Seeds of Tragopogon pratensis and Their Implications for Seed Dispersal
Source: PLoS One. 2015 May 4;10(5):e0125040. doi: 10.1371/journal.pone.0125040 (PMC4418730; doi:10.1371/journal.pone.0125040)
Supplement: S1 Appendix — (PDF) [file pone.0125040.s006.pdf]

Reynolds number

Reynolds number, *Re*, is the ratio of inertia to viscous forces. It characterizes the laminar or turbulent nature of a flow moving over a body. The definition of Reynolds number for solid bodies is given by the following equation:

$$Re = \frac{\rho V_{\infty} \, L_c}{\mu} \quad \text{(S2-1)}$$

where L_c is typically the crosswise length of the object for non-streamlined bodies, V_{∞} is the freestream velocity, ρ is the air density, and μ the air dynamic viscosity.

The estimation of the Reynolds number is a delicate issue. The characteristic length is difficult to define in the presence of the porous material that shapes the pappus.

In the attempt to calculate *Re* for *Tragopogon*, authors considered the parachute as a solid body and not as a texture of porous fibres, and this determined errors in the estimation of *Re*: Andersen (1993) defined *Re* = 990.9, whereas in Greene and Johnson (1990) it was estimated *Re* = 1.4. Following the methodology described in Rhodes (2008) and taking into account the porous texture of the fibres, Re was estimated to be around 2. For porous media, fully laminar condition exists at Reynolds numbers less than 10 and fully turbulent flow exists at Reynolds numbers greater than 2000. Hence, the flow regime is laminar for the *Re* range estimated for our parachute and all our computations were performed under this assumption.

CFD settings

In computational fluid dynamics, the interactions of a fluid with the surface of a body are described by the Navier-Stokes (N-S) equations, recalled later in Eq. (S2-3) and (S2-4). The physical bounds of the body (its geometry) need to be carefully defined to accurately reproduce these interactions. In the present case, the complex geometry of the pappus seed represented much more of a challenge: if the organization of the secondary fibres was taken into account, the complexity of the resulting model would be such that a single computation would require inadmissible computational effort, therefore the parachute has been simplified to a cone.

The geometry of the cases presented thus consists of a cone surrounded by a large domain. This wide fluid domain is required to ensure that gradients of pressure and velocity are negligeable at the outflow and that if any recirculation takes place in the wake of the cone, it will be fully inside the numerical domain and not affected by the boundaries. For both axisymmetric and 3D models the downstream length and width of the domain are equal to 18 and 11 times the diameter of the cone, respectively.

To solve the N-S equations, a system of partial differential equations, the domain then needs to be subdivided in a collection of cells called the mesh. The mesh provides a physical support to solve the N-S equations and should ideally have no influence on the solution. In that respect, a grid sensitivity analysis has been performed to assess the quality of the structured hexahedral mesh : the retained mesh exhibited neither a change in drag coefficient nor variations in pressure and velocity when further refined.

The domain is initialized in terms of velocity and pressure to the free-stream velocity and the atmospheric pressure, respectively. At the outflow, the gradient of pressure is set to zero, which is standard for such low-speed computations. Both steady and unsteady computations were launched. For

unsteady computations, the time step was set to 5 ms and a sufficient number of inner iterations is run to converge in the end of each time step.

The porous region is considered as a subdomain of the fluid domain. A sink term is added in Eq. (S2-4) to model the porous medium. Momentum loss term states that:

$$S_i = -\left(\sum_{j=1}^{3} \mathfrak{D}_{ij}\, \mu\, v_j + \sum_{j=1}^{3} C_{ij}\, \frac{\rho}{2}\, |\vec{V}| v_j\right), \forall i = \{x, y, z\} \tag{S2-2}$$

where index *i* represents each spatial dimension and index *j* is a dummy index. In this equation, the three components of the velocity vector, $\vec{V}$, are denoted by $v_j, j = \{x, y, z\}$,

There are two contributions in this equation: the contribution of viscous effects via the first term and the contribution of inertia effects via the second term. These terms can be seen as so-called Darcian and Forchheimer terms, respectively. Indices of matrices $\mathfrak{D}$ and C are useful to characterize the properties of homogeneity and isotropism of the medium. Assuming that the body is homogeneous and isotropic (e.g. constant permeability along the ribs), then only diagonal components of matrices are non-zero.

Inertia effects can be ignored in the present situation due to the fact that the flow is laminar (Rhodes 2008). Hence, matrix *C* is equal to zero. It remains matrix $\mathfrak{D}$ that is function of permeability. Permeability therefore is the only coefficient that is useful to adjust.

Navier-Stokes equations, were solved using Ansys v13.0 commercial software. Ansys ICEM enabled us to build the geometry and the mesh. Ansys CFX was used as a solver and also for pre- and post-processing. CFD simulations were run on a single computer with a 3.30 GHz Quad-core processor (32bit) with 4.0 GB of RAM.

Computation of drag coefficient from the simulation

The Navier-Stokes equations are first recalled hereafter for an incompressible fluid

Continuity equation : $$\frac{\partial v_j}{\partial x_j} = 0 \qquad \text{(S2-3)}$$

and

Momentum equations : $$\frac{\partial v_i}{\partial t} + v_j \frac{\partial v_i}{\partial x_j} - \frac{1}{\rho}\frac{\partial \sigma_{ij}}{\partial x_j} - f_i = 0 \ , \forall i = \{x, y, z\} \qquad \text{(S2-4)}$$

From left to right in the momentum equations, the terms denote the effects of the

- time rate of change of control volume momentum
- net flux of momentum leaving a control volume (convection term)
- surface forces acting on a control surface
- body forces acting on a control volume.

After some simplifications, the drag force, F_{D}, (which belongs to surface forces) is obtained by projection of the resulting force on the x-axis. The CFD software computes it. The drag coefficient is then deduced from:

$$C_{\mathrm{D}} = \frac{F_{\mathrm{D}}}{\frac{1}{2}\rho\ \mathrm{V}_{\infty}^{2} A} \qquad \text{(S2-5)}$$

where *A* is the cross-sectional area of the object.
