# Supplementary material for: Morphologic and Aerodynamic Considerations Regarding the Plumed Seeds of Tragopogon pratensis and Their Implications for Seed Dispersal
Source: PLoS One. 2015 May 4;10(5):e0125040. doi: 10.1371/journal.pone.0125040 (PMC4418730; doi:10.1371/journal.pone.0125040)
Supplement: S2 Appendix — (PDF) [file pone.0125040.s007.pdf]

List of abbreviations

Quantity	Unit	Meaning
A	m^2	cross-sectional area / pappus area
C_D		drag coefficient
d	m	diameter of the hairs
D	m	diameter of the ribs, as a function of D_I and D_{III}
D_I, D_{II}, D_{III}	m	diameters of the ribs measured in the first, second, and third regions from the centre
E		porosity of the pappus
k		Kozeny factor
K	m^2	permeability
l	m	total length of the hairs
L	m	total length of the ribs
m	mg	mass of the parachute
Re		Reynolds number
S_{cone}	m^2	surface of the pappus modelled by a cone
S_{hairs}	m^2	surface occupied by the hairs
S_{ribs}	m^2	surface occupied by the ribs
S_v	m^{-1}	fibre surface area per unit volume of fibre
V_∞	$m \cdot s^{-1}$	rate of descent / free-stream velocity / speed of the object relative to the fluid
ϱ	$kg \cdot m^{-3}$	air density
