# Supplementary material for: Morphologic and Aerodynamic Considerations Regarding the Plumed Seeds of Tragopogon pratensis and Their Implications for Seed Dispersal
Source: PLoS One. 2015 May 4;10(5):e0125040. doi: 10.1371/journal.pone.0125040 (PMC4418730; doi:10.1371/journal.pone.0125040)
Supplement: S1 Datasets — (ZIP) [file pone.0125040.s008.zip › Morphological analysis/ Morphological analysis of seeds.pdf]

# Seed morphology

| | Tot Mass (mg) | Parachute mass | Achene mass | % | N. of ribs | Rib diameter (D) | | |
|---|---|---|---|---|---|---|---|---|
| | | | | | | 0-5 mm | 5-10 mm | 10-15 mm |
| **Peripheral seeds** | 12.74 | 3 | 9.74 | 0.24 | 28 | 76.73 | 67.02 | 67.58 |
| | 10.53 | 1.6 | 8.93 | 0.15 | 25 | 90.19 | 78.76 | 75.60 |
| | 12.48 | 2.3 | 10.18 | 0.18 | 28 | 115.11 | 93.94 | 87.30 |
| | 11.05 | 2.2 | 8.85 | 0.20 | 29 | 121.08 | 106.94 | 88.91 |
| | 11.57 | 2.8 | 8.77 | 0.24 | 24 | 91.10 | 79.36 | 59.56 |
| | 10.79 | 1.8 | 8.99 | 0.17 | 28 | 118.46 | 112.22 | 100.23 |
| | 11.83 | 1.6 | 10.23 | 0.14 | 29 | 121.01 | 115.05 | 81.56 |
| | 10.14 | 1.7 | 8.44 | 0.17 | 30 | 97.41 | 86.59 | 71.29 |
| | 10.79 | 1.3 | 9.49 | 0.12 | 28 | 91.69 | 86.52 | 75.64 |
| | | | | | 28 | | | |

| | Tot Mass (mg) | Parachute mass | Achene mass | % | N. of ribs | Rib diameter (D) | | |
|---|---|---|---|---|---|---|---|---|
| | | | | | | 0-5 mm | 5-10 mm | 10-15 mm |
| **Central seeds** | 8.06 | 1.7 | 6.36 | 0.21 | 27 | 125.31 | 101.66 | 80.37 |
| | 7.93 | 1.7 | 6.23 | 0.21 | 25 | 111.81 | 81.35 | 81.41 |
| | 8.58 | 2.1 | 6.48 | 0.24 | 26 | 96.03 | 96.00 | 85.47 |
| | 6.76 | 1.4 | 5.36 | 0.21 | 28 | 102.03 | 102.23 | 87.30 |
| | 7.54 | 2 | 5.54 | 0.27 | 29 | 126.46 | 108.52 | 94.19 |
| | 8.19 | 2.5 | 5.69 | 0.31 | 28 | 74.14 | 69.64 | 56.35 |
| | 8.06 | 2.4 | 5.66 | 0.30 | 26 | 99.25 | 70.51 | 60.64 |
| | 7.67 | 1.8 | 5.87 | 0.23 | 29 | 110.98 | 110.29 | 101.12 |
| | 8.06 | 2.3 | 5.76 | 0.29 | 28 | 102.96 | 90.56 | 79.36 |
| | | | | | 29 | | | |
