# Supplementary material for: Morphologic and Aerodynamic Considerations Regarding the Plumed Seeds of Tragopogon pratensis and Their Implications for Seed Dispersal
Source: PLoS One. 2015 May 4;10(5):e0125040. doi: 10.1371/journal.pone.0125040 (PMC4418730; doi:10.1371/journal.pone.0125040)
Supplement: S1 Datasets — (ZIP) [file pone.0125040.s008.zip › Morphological analysis/porosity.pdf]

Porosity calculated only on secondary hairs

Images from inverted microscope (each column 1 image) from 10 seeds

$$E_{hairs} = \frac{A_{white\ px}}{A_{total\ px}}$$

Calbration px/um2 0.238144

0-5mm			5-10 mm			10-15mm		
A tot px	A white px	E hairs	A tot px	A white px	Porosity	A tot px	A white px	Porosity
1015589	965977	0.95115	1116224	1013873	0.908306	1156828	1099500	0.9504438
640974	601783	0.938857	787718	778171	0.9878802	383219	367419	0.9587703
819590	757970	0.924816	224624	222244	0.9894045	590873	576030	0.9748795
689517	670350	0.972202	1293006	1227875	0.9496282	369860	353375	0.9554291
284897	263754	0.925787	1184028	1123480	0.9488627	980809	924167	0.9422497
833620	805982	0.966846	1074422	1024161	0.9532204	985699	941413	0.9550715
528776	516106	0.976039	189262	182597	0.9647843	865253	821315	0.9492195
582601	564708	0.969288	1193129	1132736	0.9493827	785155	735172	0.93634
543795	530681	0.975884	1204830	1155166	0.9587792	1075491	1040868	0.9678073
588540	570613	0.96954	1161940	1110177	0.9554512	792866	763893	0.9634579
109344	104612	0.956724	808055	796457	0.985647	1233695	1172988	0.9507925
728319	680501	0.934345	275176	270705	0.9837522	815292	784916	0.9627422
565636	543932	0.961629	569249	527746	0.9270917	561618	526041	0.9366527
264266	254406	0.962689	1096358	1015854	0.9265714	902062	862568	0.9562181
236256	229398	0.970972	881878	824335	0.9347495	647993	632499	0.9760892
**Mean**	**0-5 mm**	**0.957118**	**Mean**	**5-10 mm**	**0.9549008**	**Mean**	**10-15 mm**	**0.9557442**
**SE**		**0.004624**	**SE**		**0.0063319**	**SE**		**0.0031338**
