# Supplementary figures and images for: Morphologic and Aerodynamic Considerations Regarding the Plumed Seeds of Tragopogon pratensis and Their Implications for Seed Dispersal
Source: PLoS One. 2015 May 4;10(5):e0125040. doi: 10.1371/journal.pone.0125040 (PMC4418730; doi:10.1371/journal.pone.0125040)

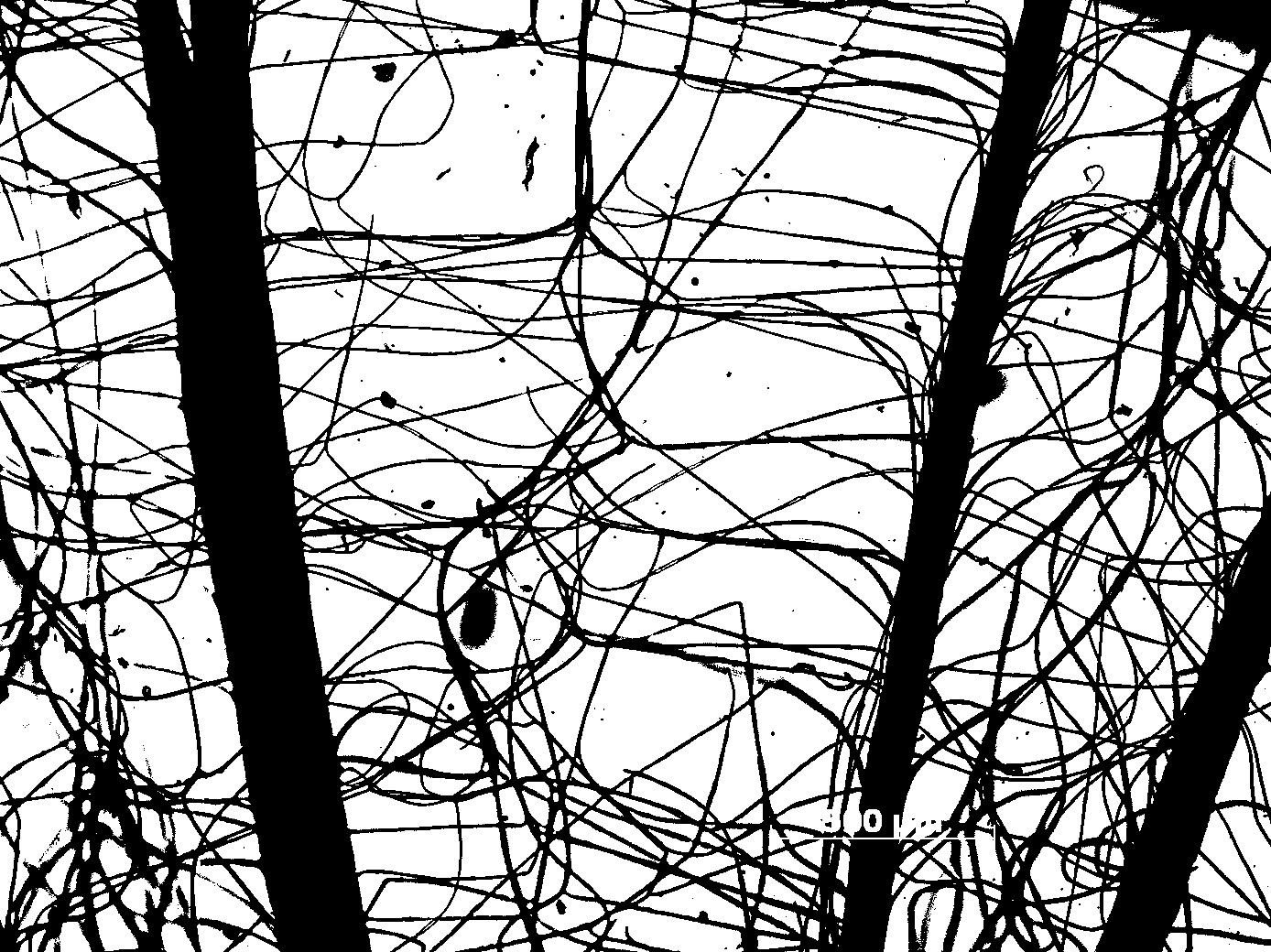

Supplement: S1 Datasets — (ZIP) [file pone.0125040.s008.zip › Morphological analysis/BW image - 0-5 mm/SNAP-101607-0001 BW.jpg]

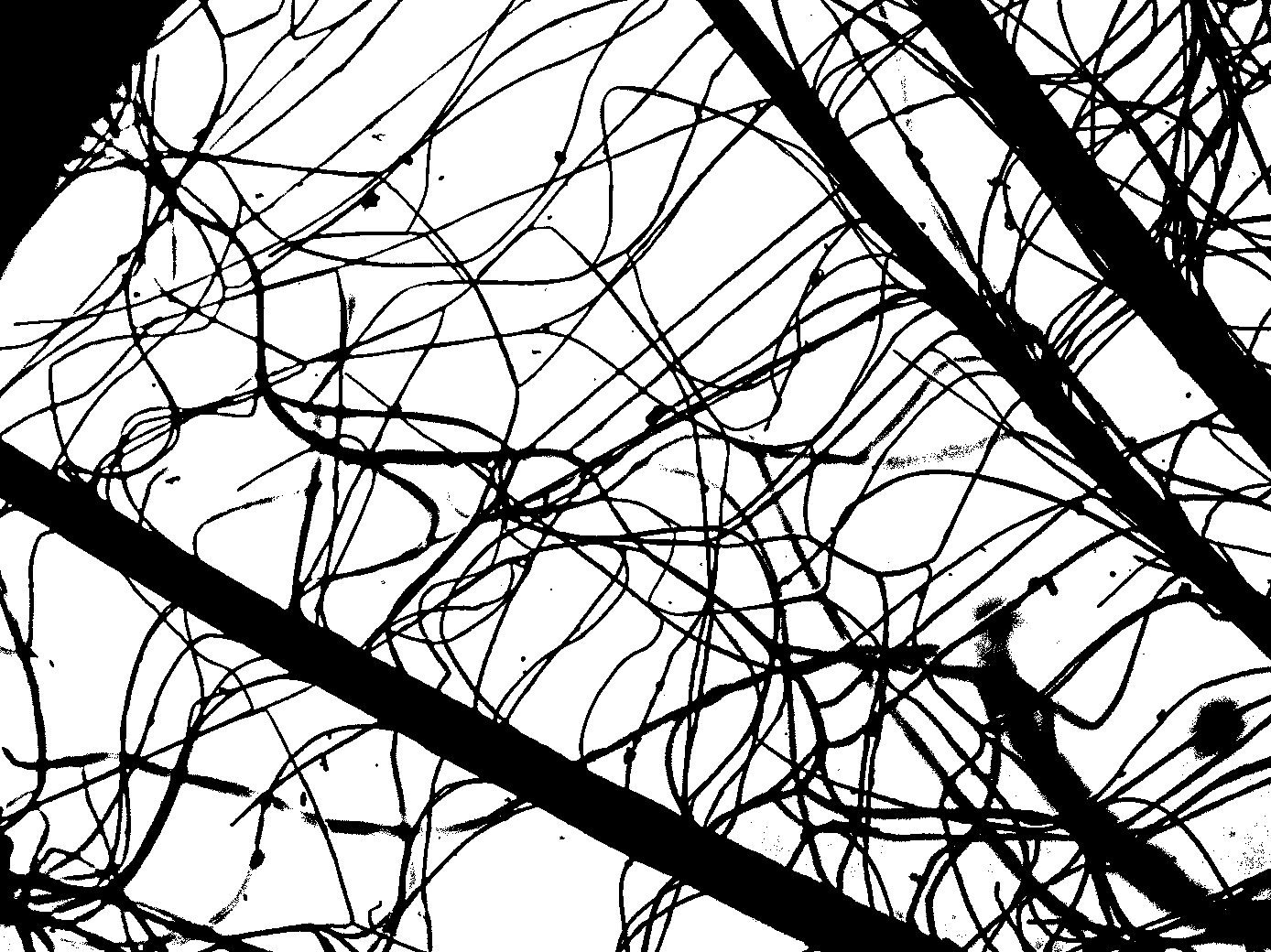

Supplement: S1 Datasets — (ZIP) [file pone.0125040.s008.zip › Morphological analysis/BW image - 0-5 mm/SNAP-101705-0003 BW.jpg]

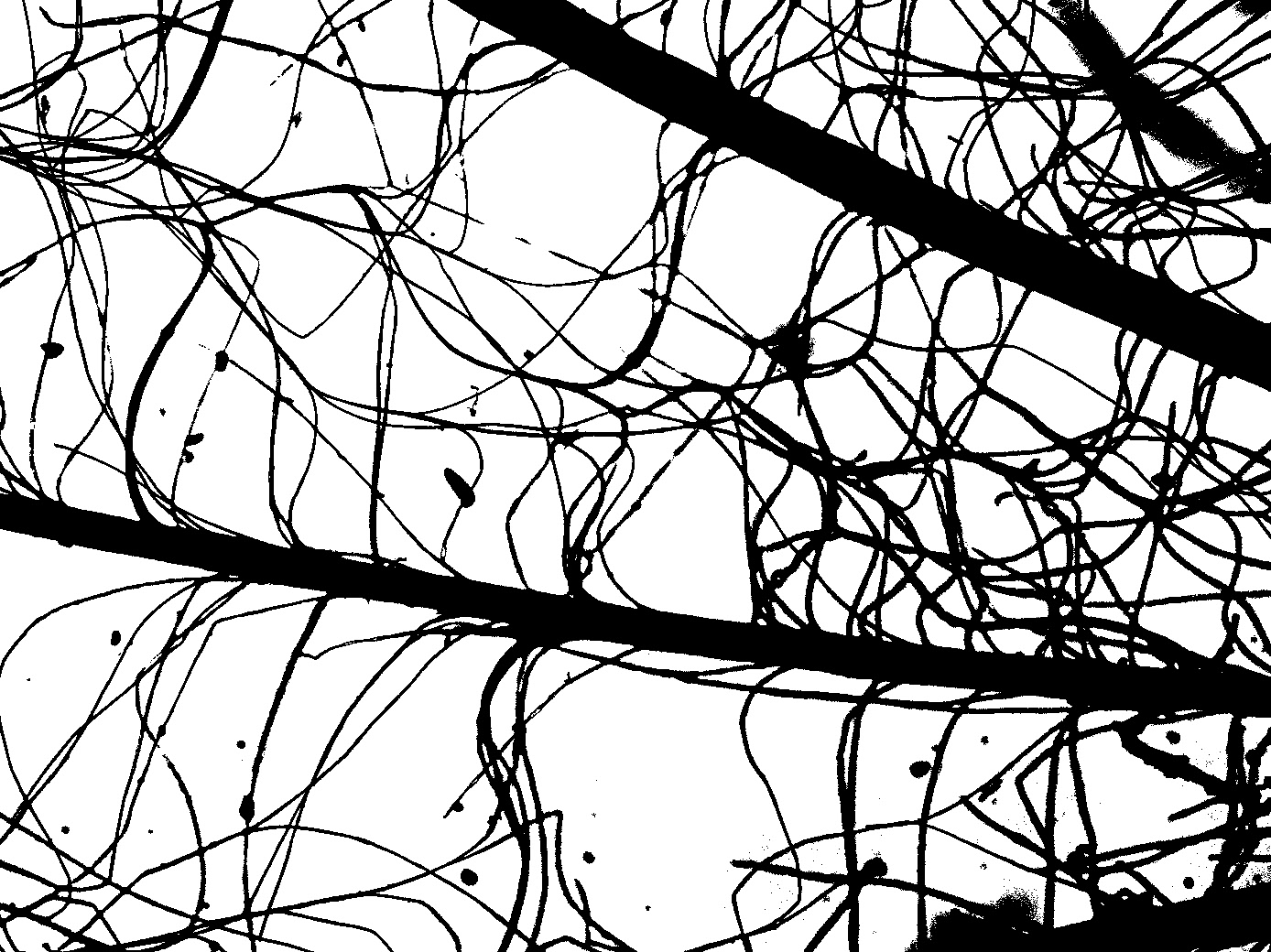

Supplement: S1 Datasets — (ZIP) [file pone.0125040.s008.zip › Morphological analysis/BW image - 0-5 mm/SNAP-101717-0004 BW.jpg]

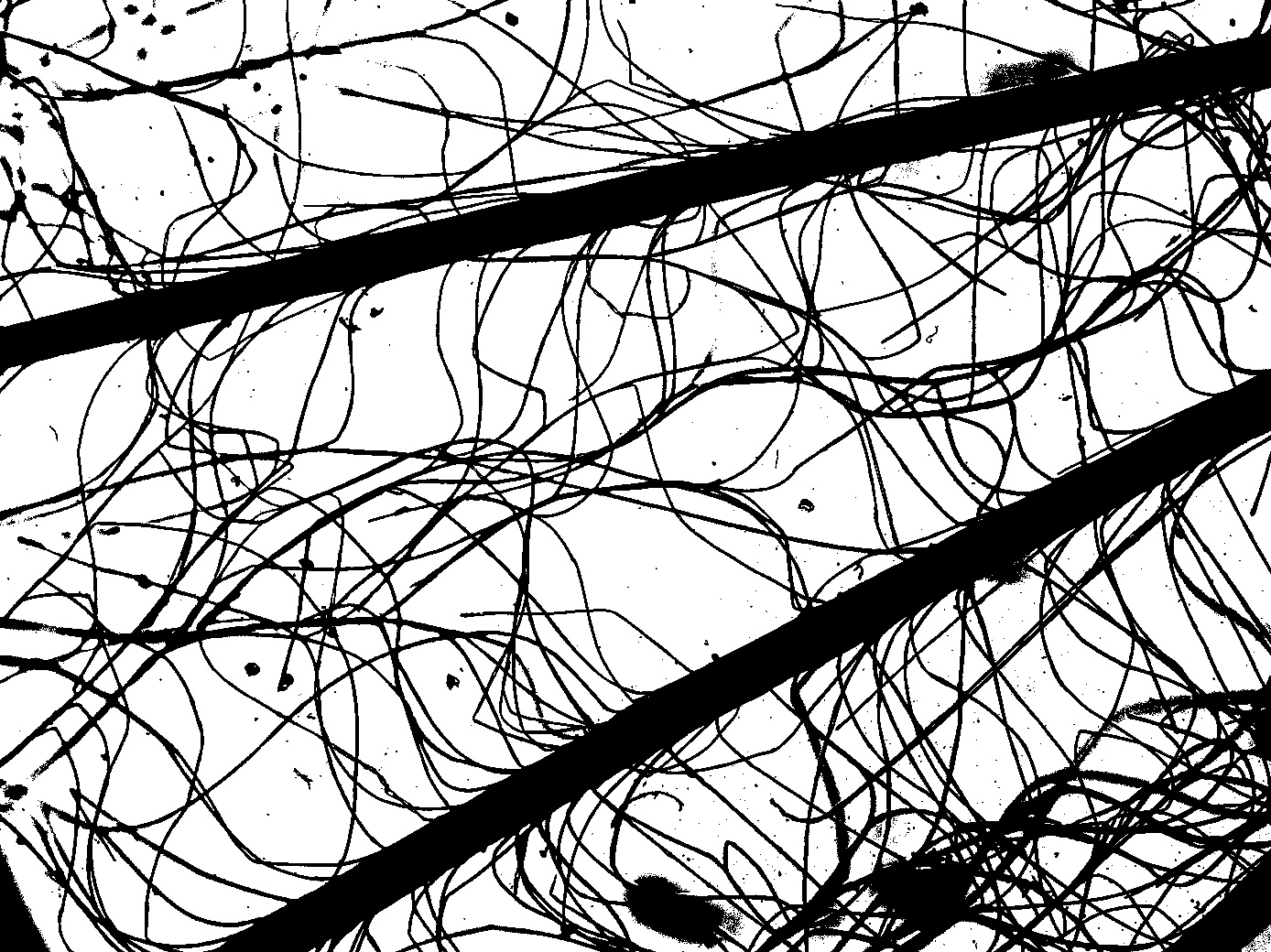

Supplement: S1 Datasets — (ZIP) [file pone.0125040.s008.zip › Morphological analysis/BW image - 0-5 mm/SNAP-101731-0005 BW.jpg]

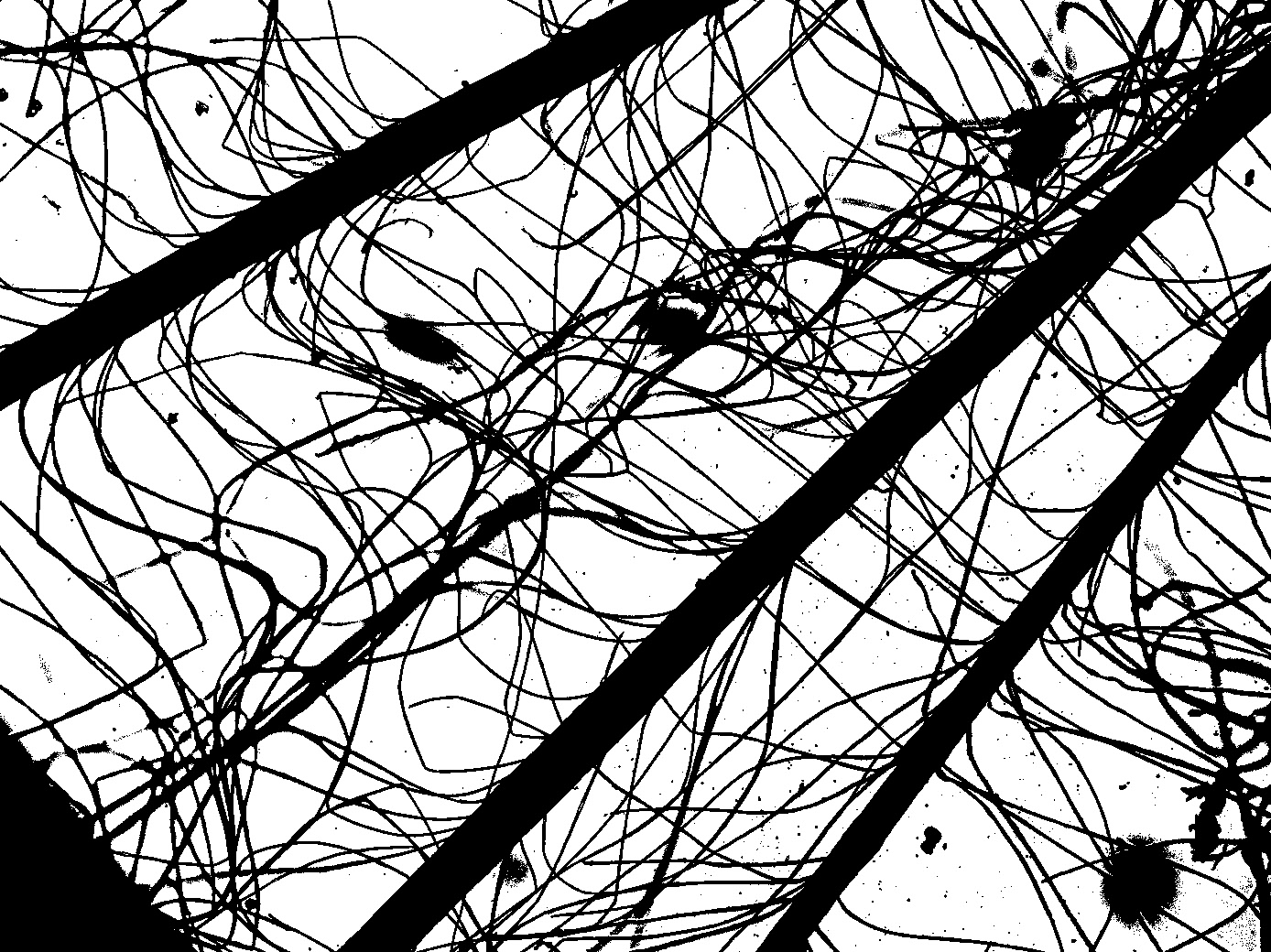

Supplement: S1 Datasets — (ZIP) [file pone.0125040.s008.zip › Morphological analysis/BW image - 0-5 mm/SNAP-101743-0006 BW.jpg]

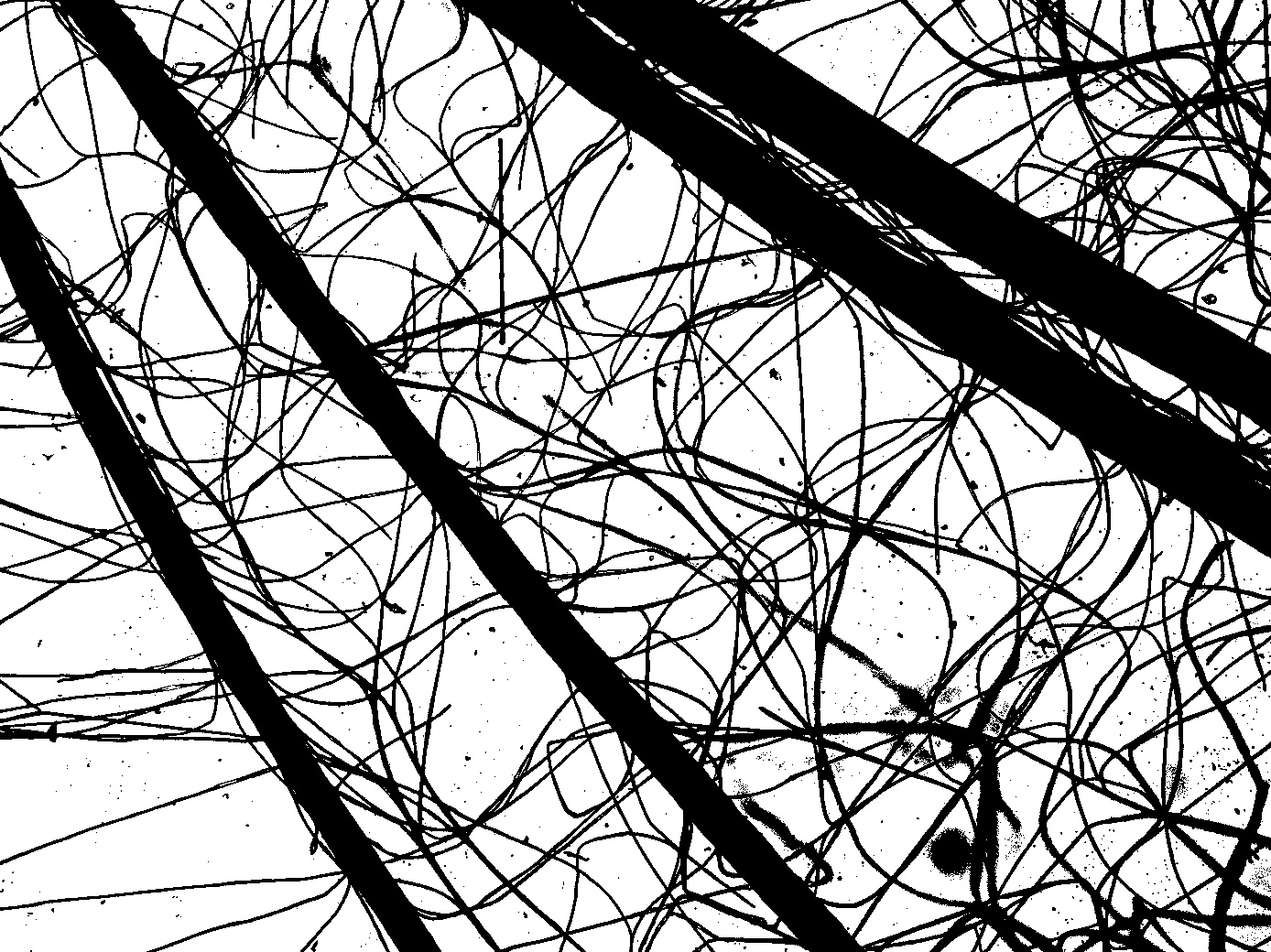

Supplement: S1 Datasets — (ZIP) [file pone.0125040.s008.zip › Morphological analysis/BW image - 0-5 mm/SNAP-101822-0009 BW.jpg]

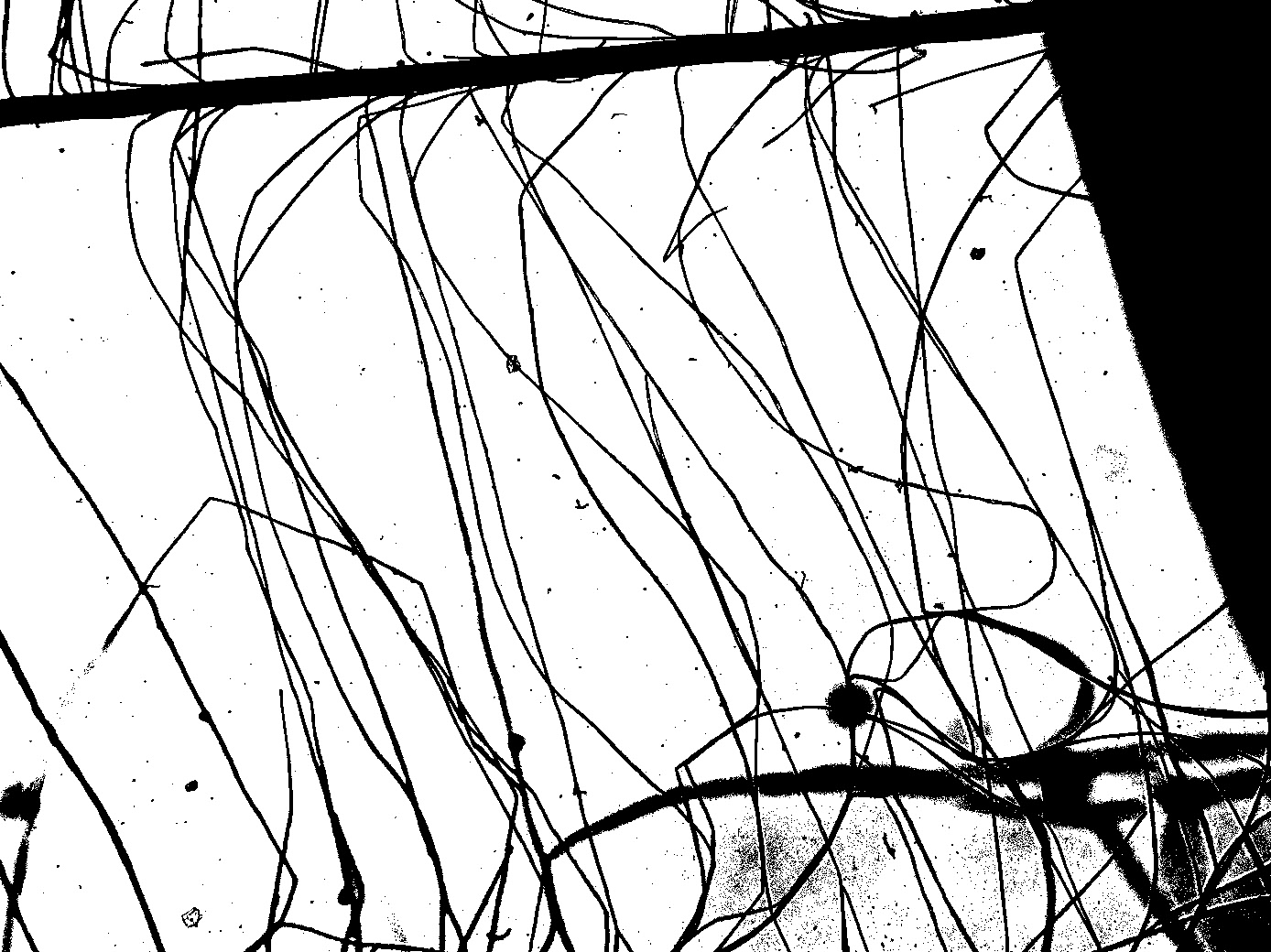

Supplement: S1 Datasets — (ZIP) [file pone.0125040.s008.zip › Morphological analysis/BW image - 10-15 mm/SNAP-104324-0038 BW.jpg]

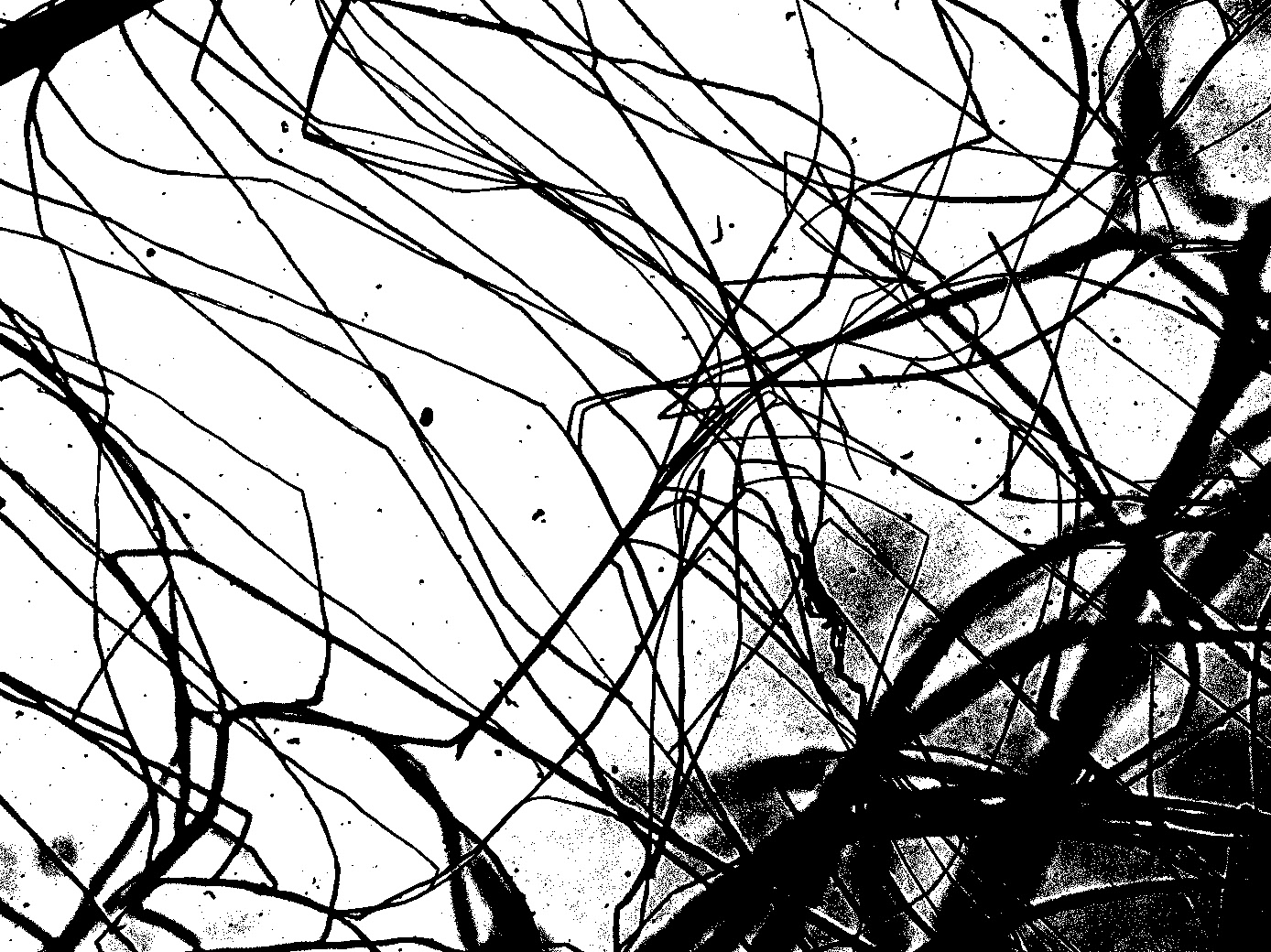

Supplement: S1 Datasets — (ZIP) [file pone.0125040.s008.zip › Morphological analysis/BW image - 10-15 mm/SNAP-104342-0039 BW.jpg]

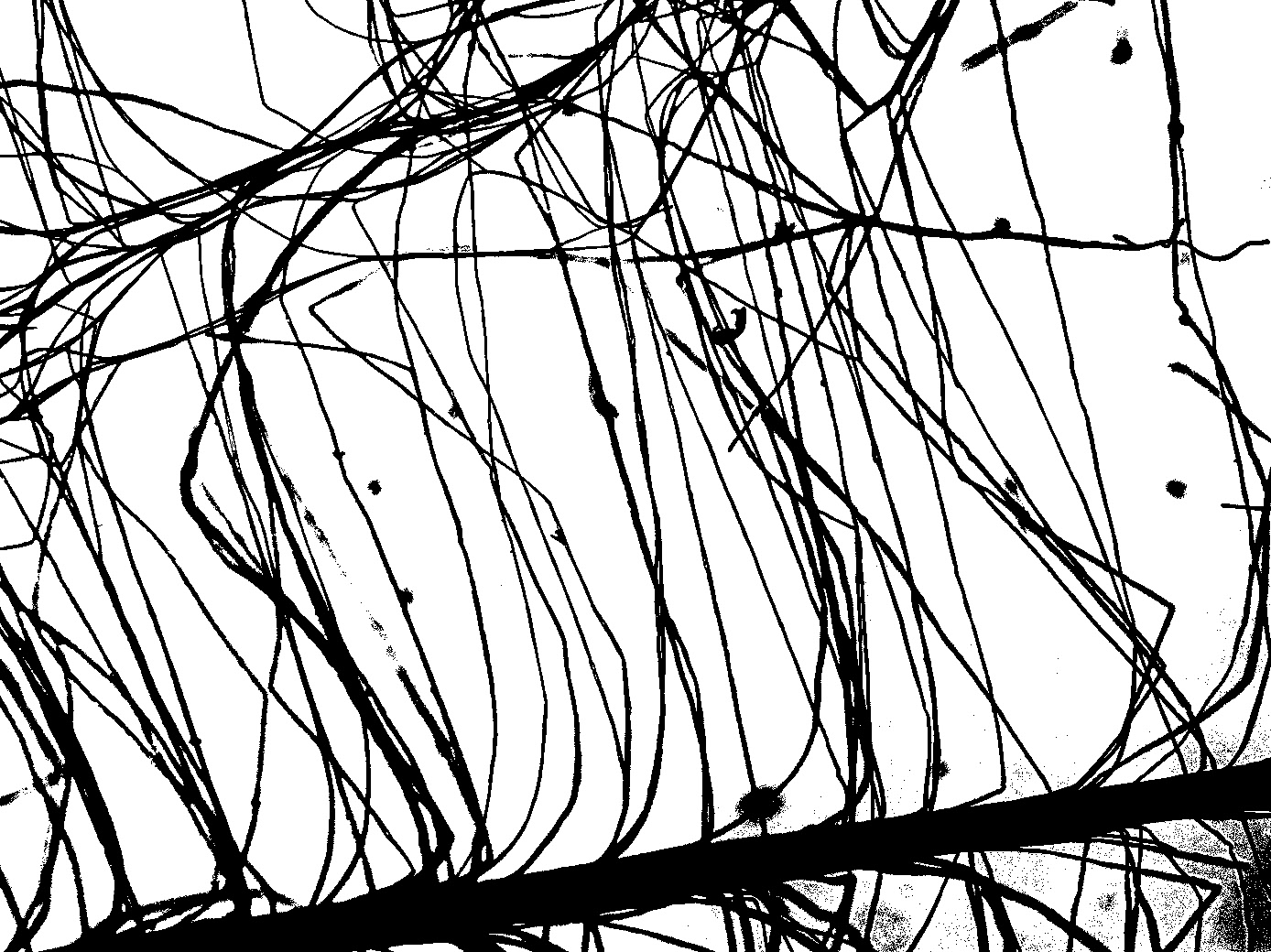

Supplement: S1 Datasets — (ZIP) [file pone.0125040.s008.zip › Morphological analysis/BW image - 10-15 mm/SNAP-104427-0040 BW.jpg]

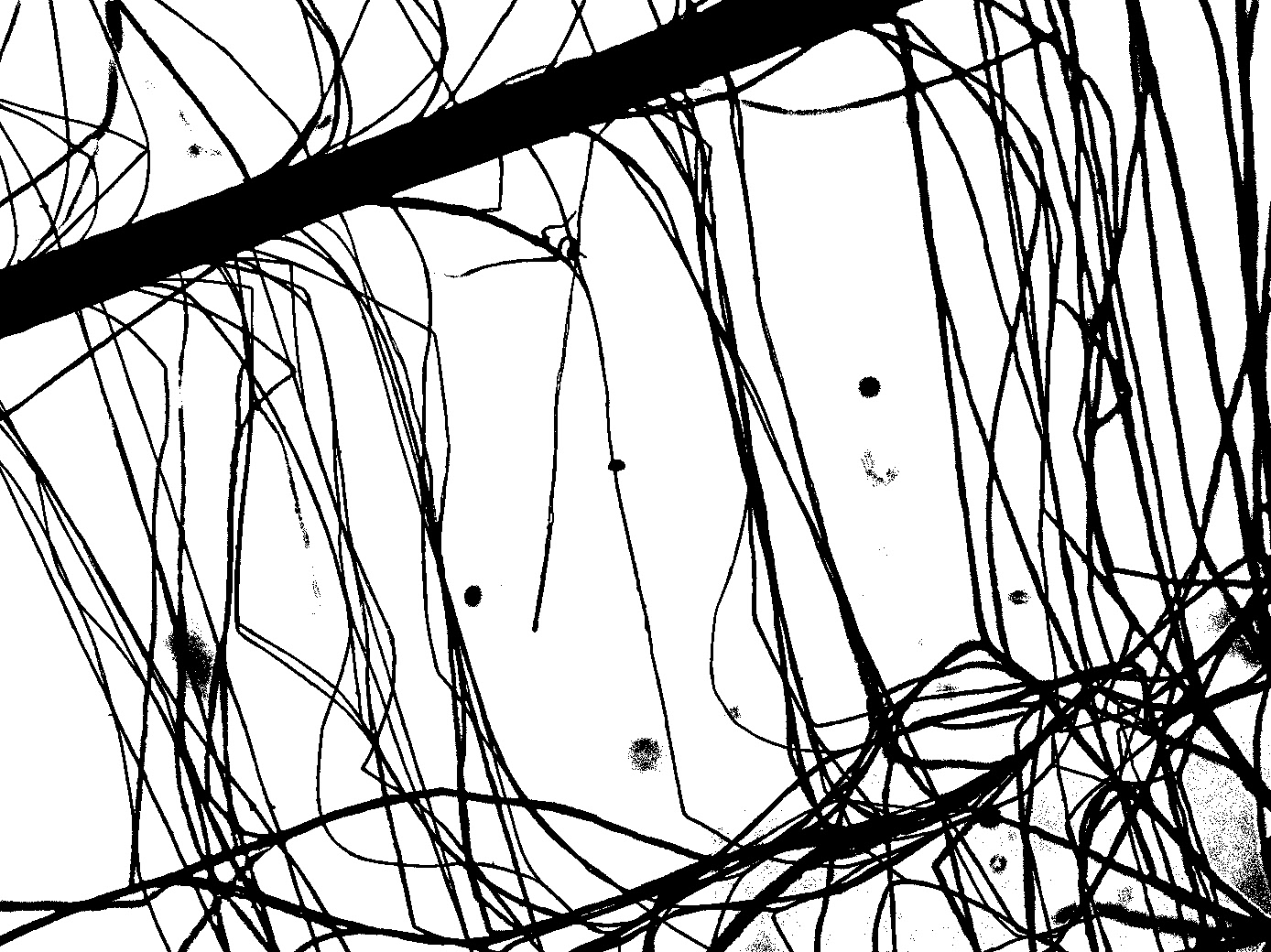

Supplement: S1 Datasets — (ZIP) [file pone.0125040.s008.zip › Morphological analysis/BW image - 10-15 mm/SNAP-104435-0041 BW.jpg]

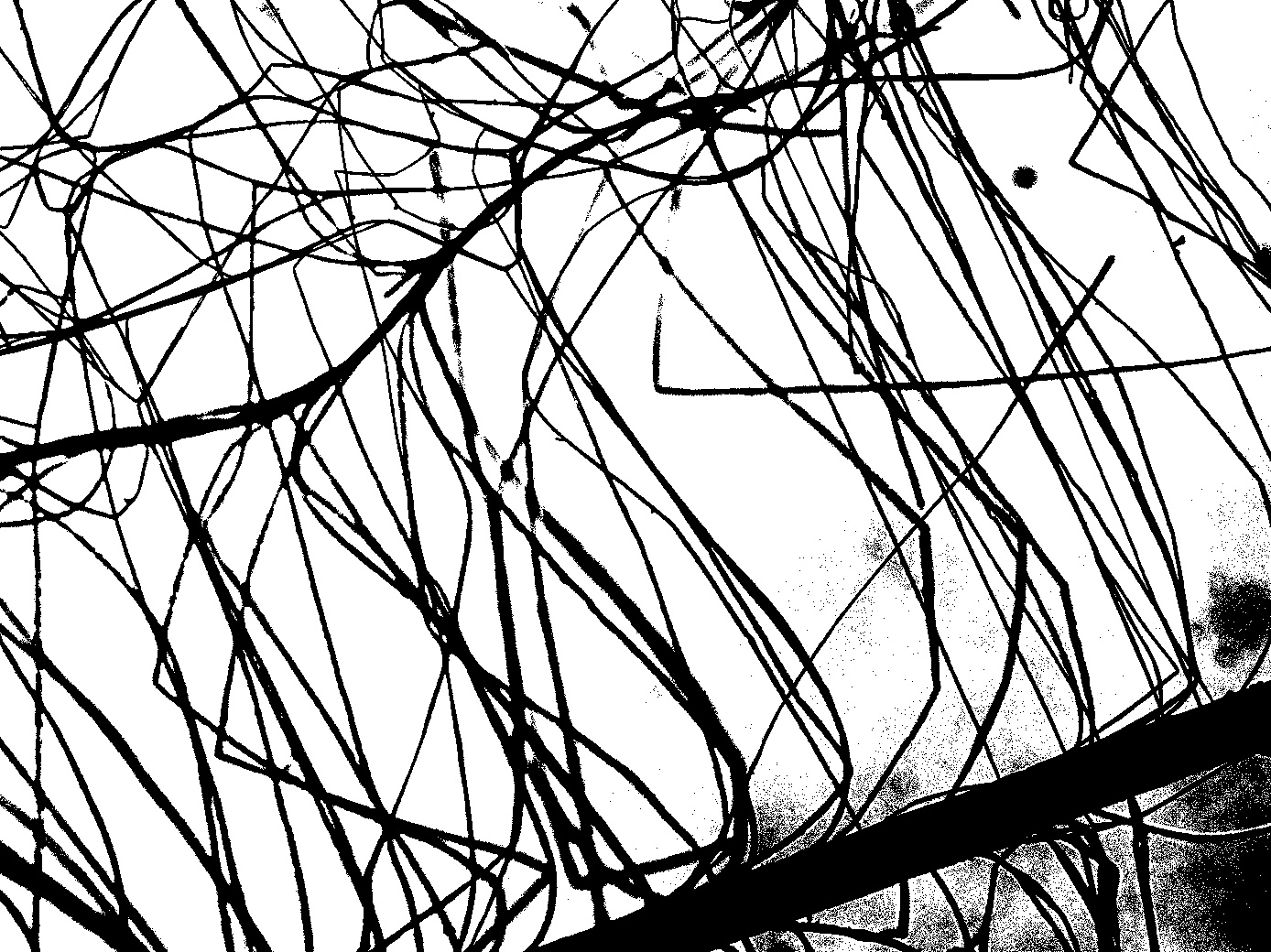

Supplement: S1 Datasets — (ZIP) [file pone.0125040.s008.zip › Morphological analysis/BW image - 10-15 mm/SNAP-104446-0042 BW.jpg]

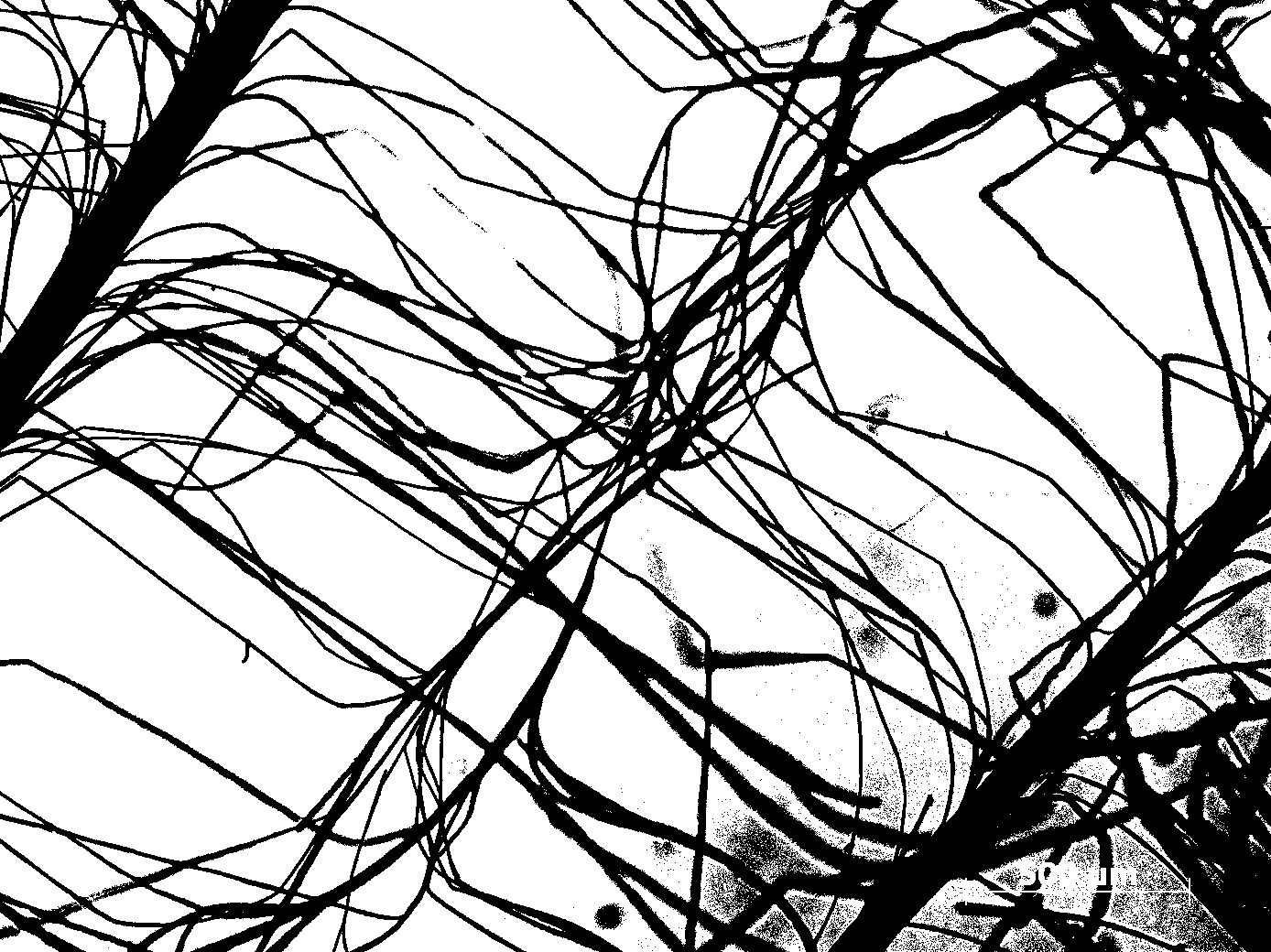

Supplement: S1 Datasets — (ZIP) [file pone.0125040.s008.zip › Morphological analysis/BW image - 10-15 mm/SNAP-104510-0044 BW.jpg]

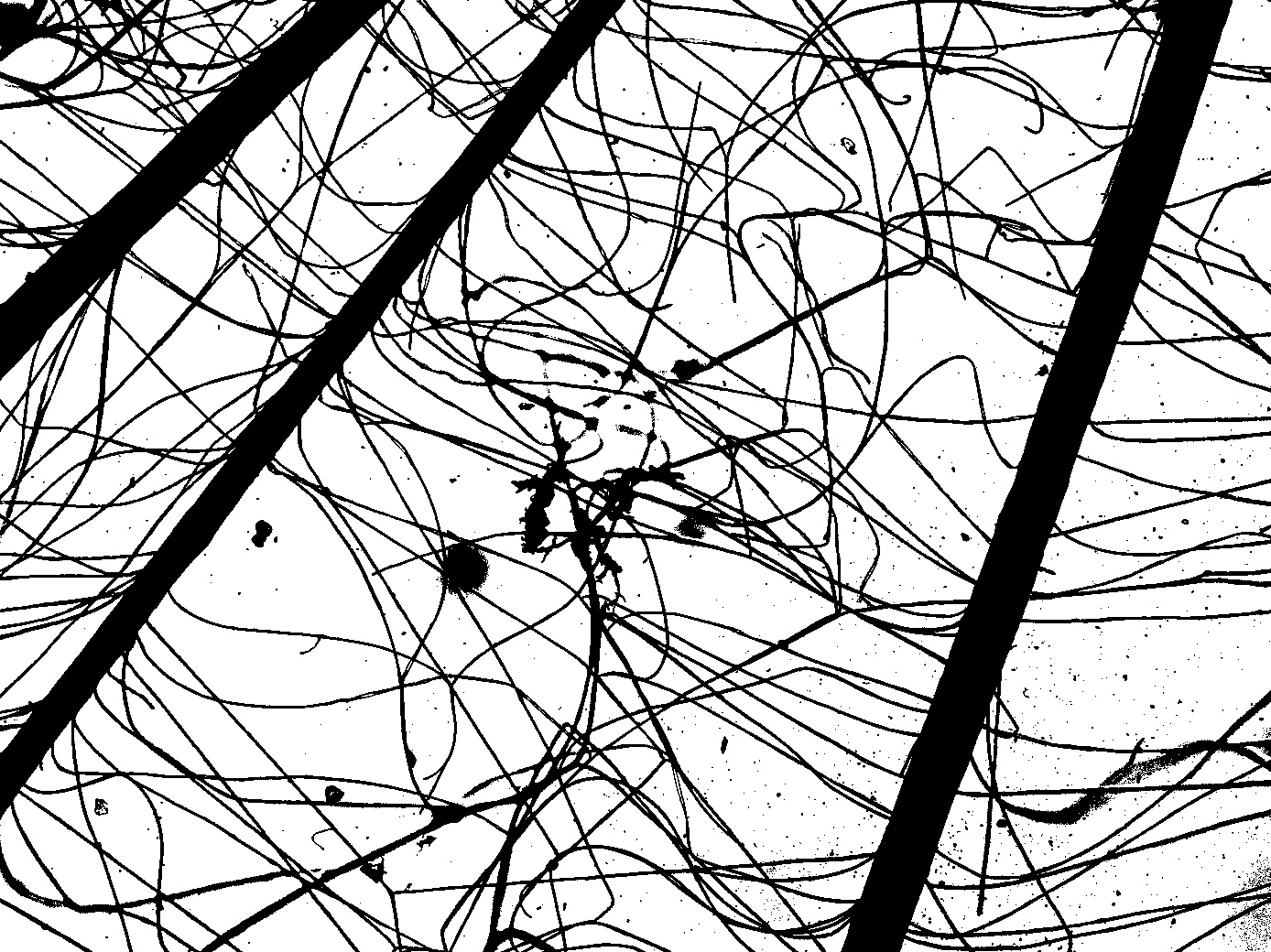

Supplement: S1 Datasets — (ZIP) [file pone.0125040.s008.zip › Morphological analysis/BW image - 5-10 mm/SNAP-101753-0007 BW.jpg]

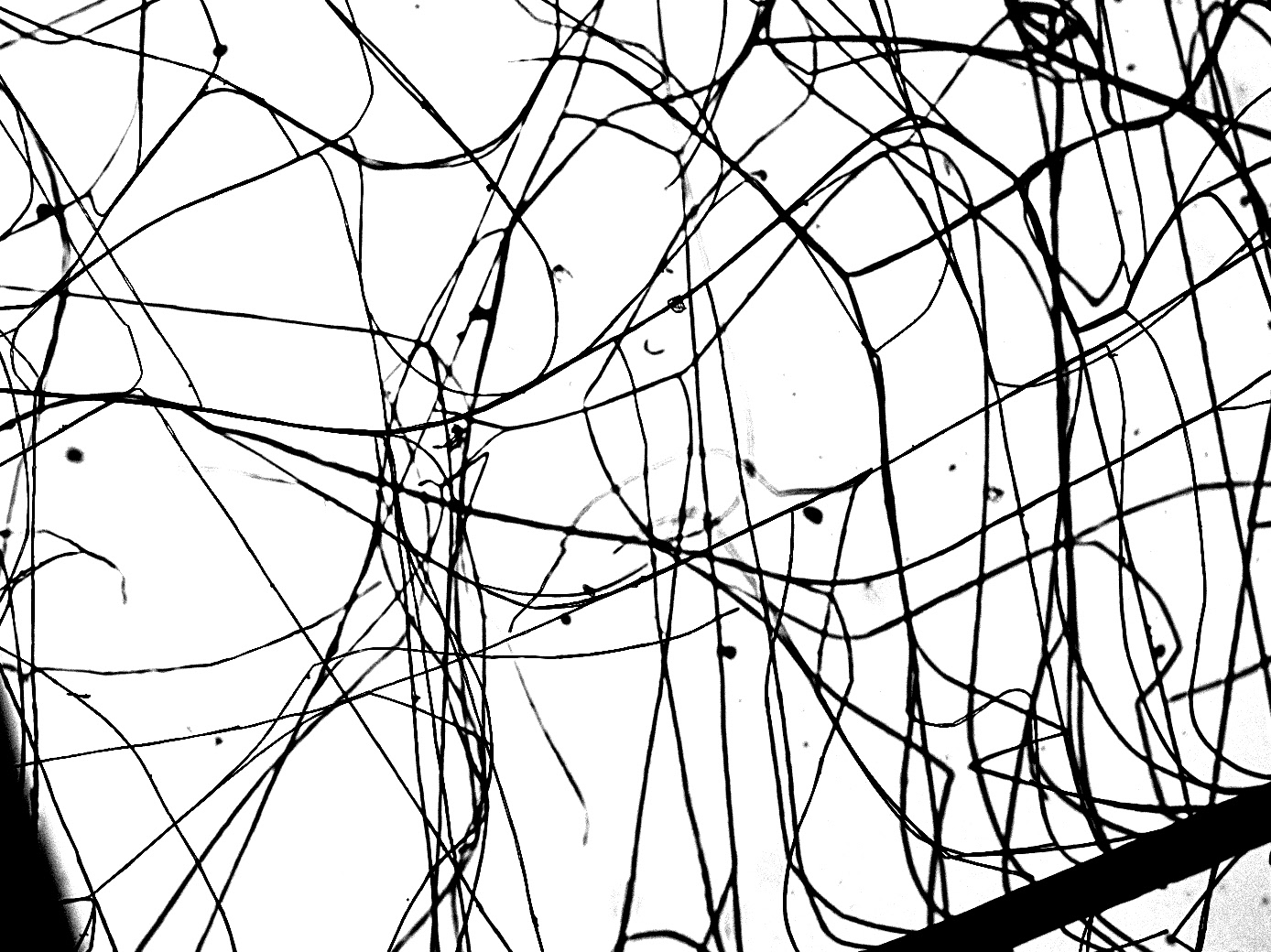

Supplement: S1 Datasets — (ZIP) [file pone.0125040.s008.zip › Morphological analysis/BW image - 5-10 mm/SNAP-110037-0057.jpg]

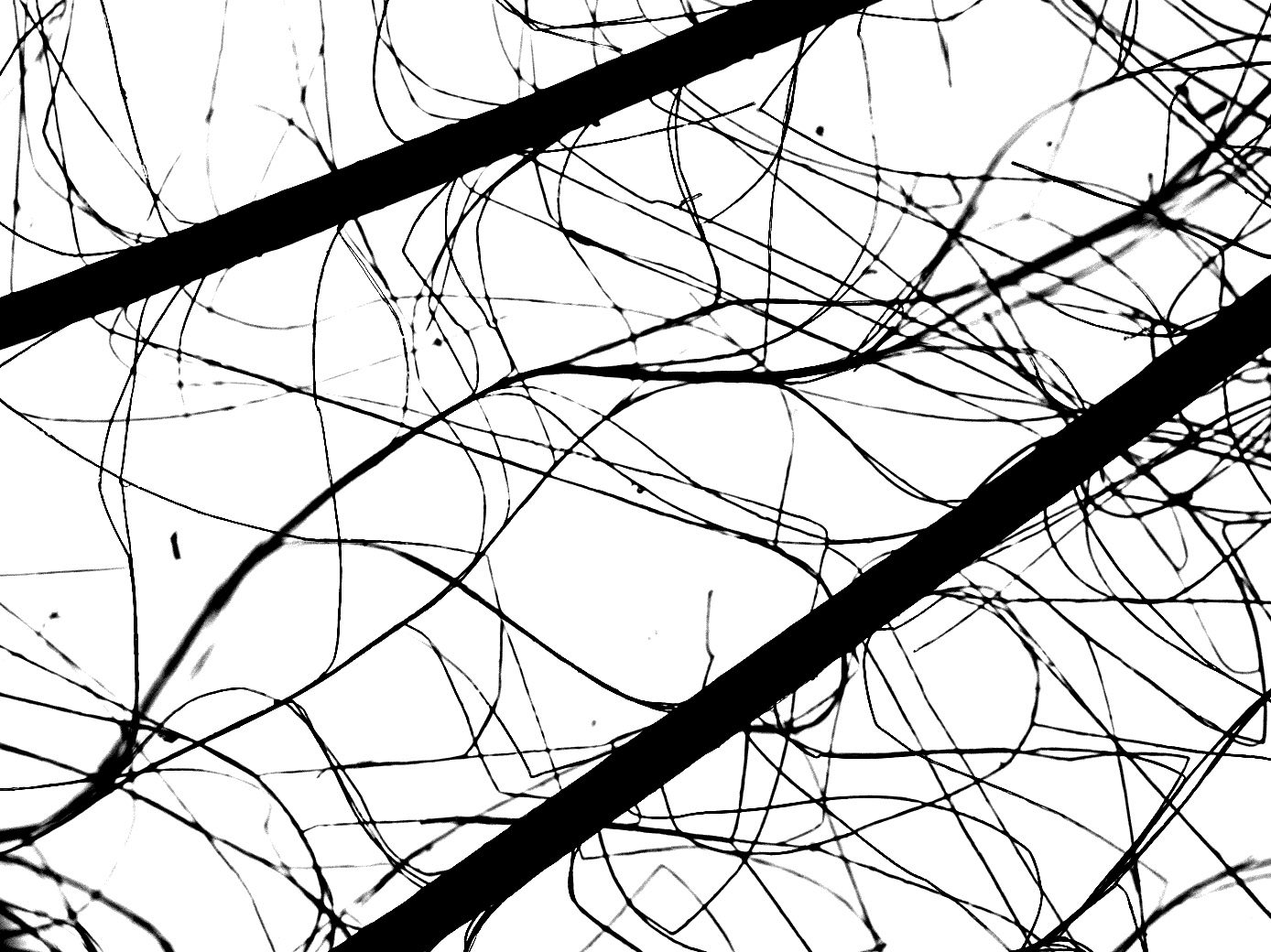

Supplement: S1 Datasets — (ZIP) [file pone.0125040.s008.zip › Morphological analysis/BW image - 5-10 mm/SNAP-110052-0058.jpg]

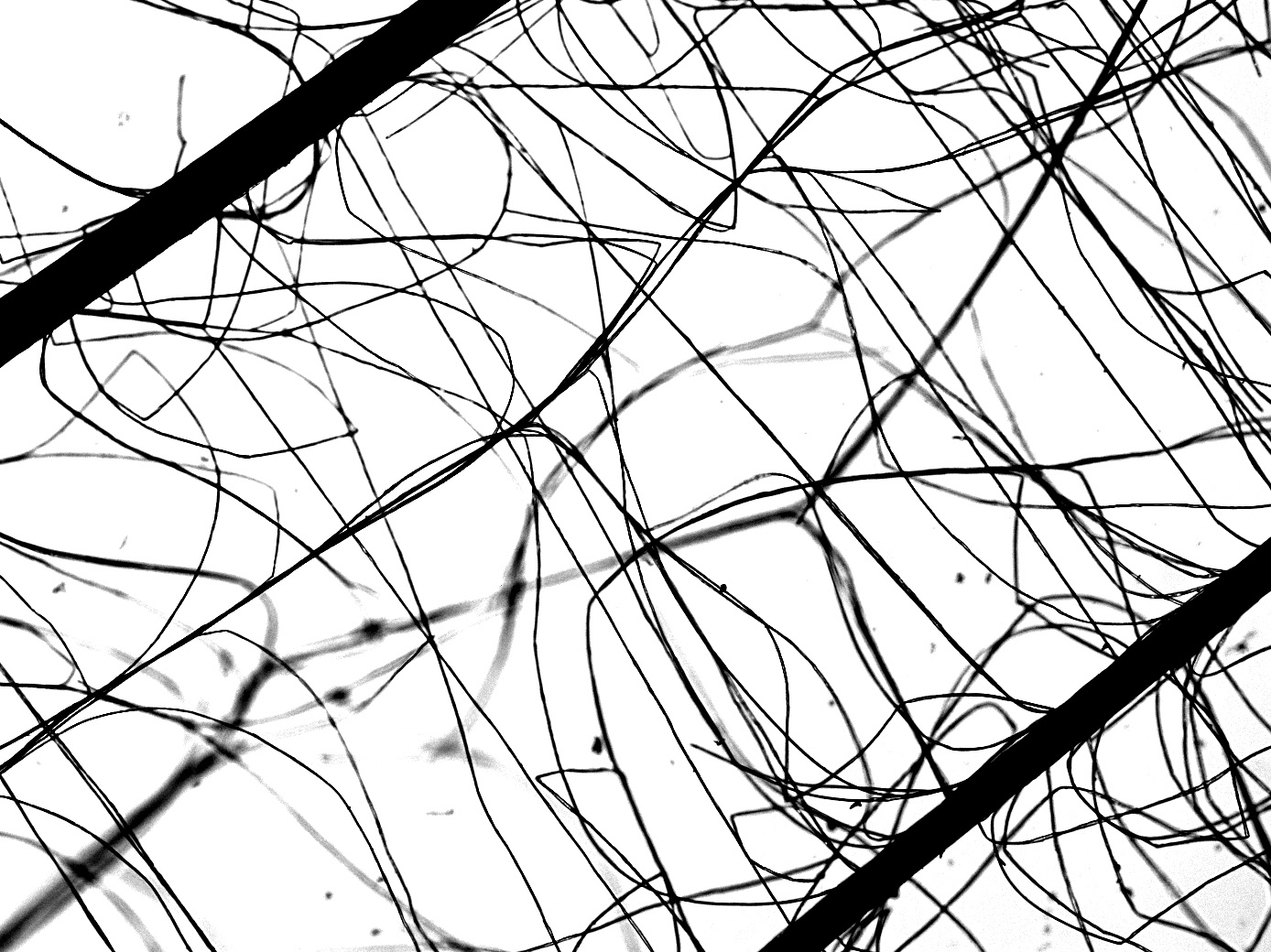

Supplement: S1 Datasets — (ZIP) [file pone.0125040.s008.zip › Morphological analysis/BW image - 5-10 mm/SNAP-110106-0059.jpg]

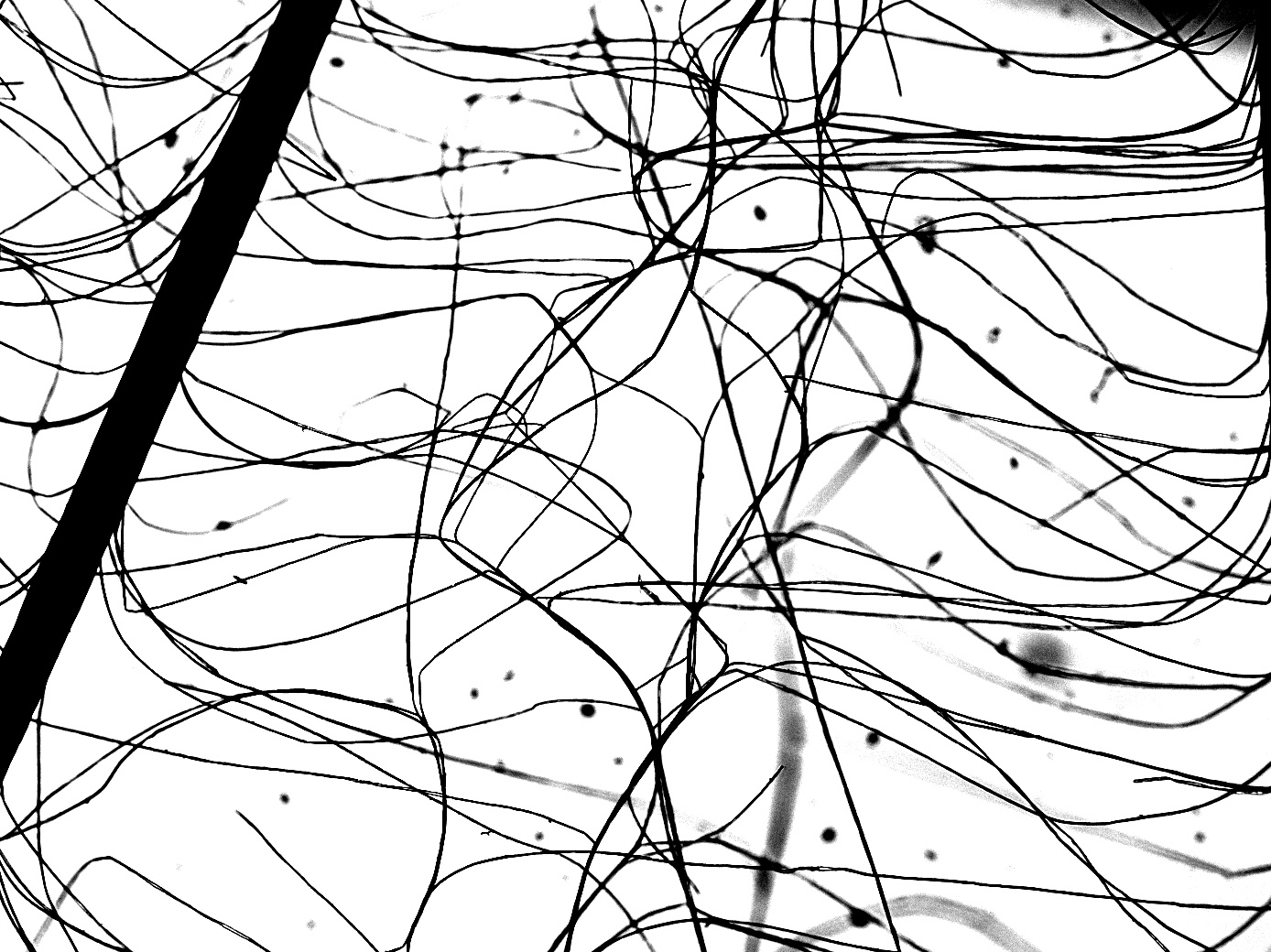

Supplement: S1 Datasets — (ZIP) [file pone.0125040.s008.zip › Morphological analysis/BW image - 5-10 mm/SNAP-110128-0061.jpg]

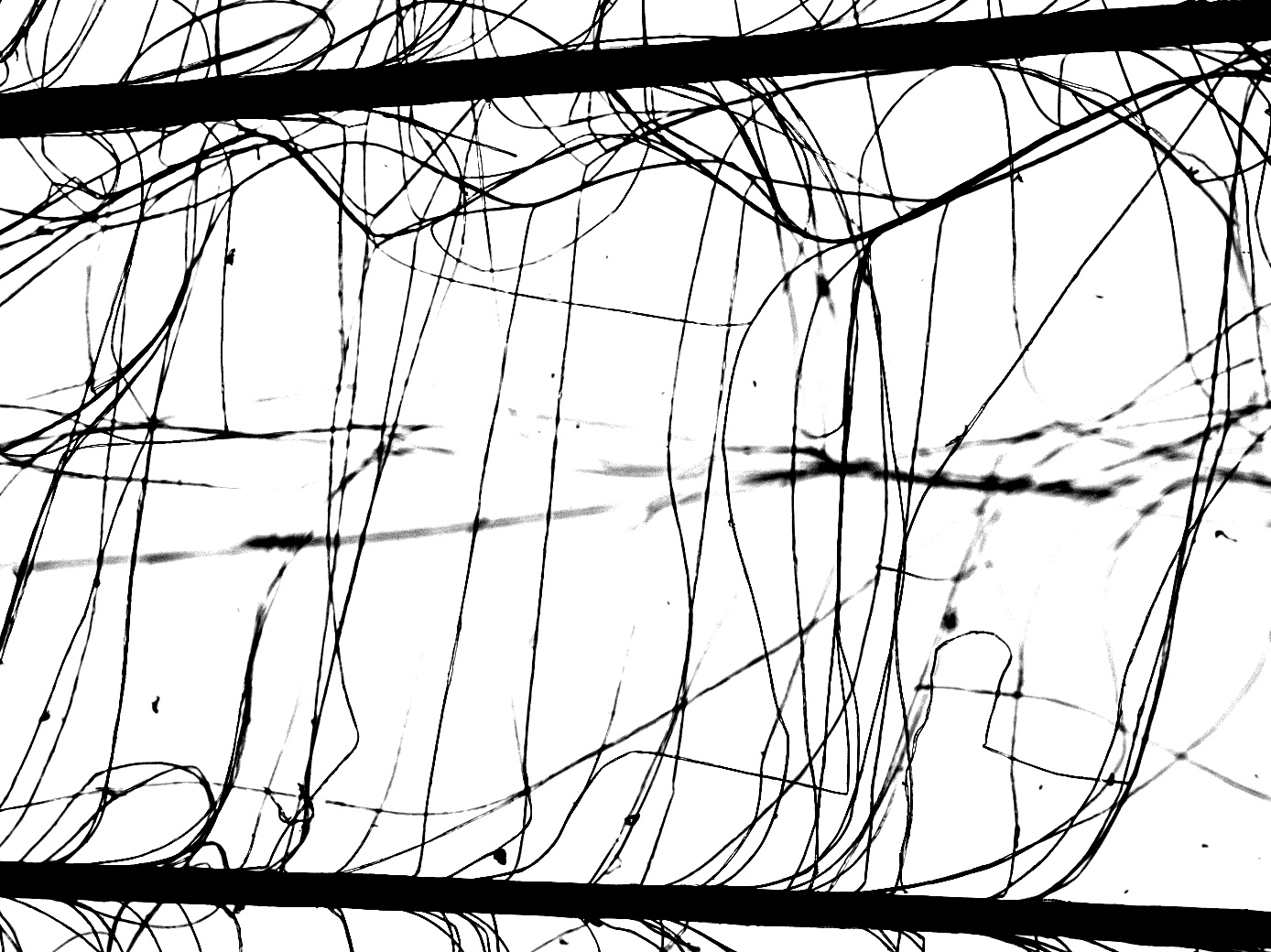

Supplement: S1 Datasets — (ZIP) [file pone.0125040.s008.zip › Morphological analysis/BW image - 5-10 mm/SNAP-110217-0065.jpg]
